# Autistic adults have poorer quality healthcare and worse health based on self-report data

**DOI:** 10.1186/s13229-022-00501-w

**Published:** 2022-05-26

**Authors:** Elizabeth Weir, Carrie Allison, Simon Baron-Cohen

**Affiliations:** Department of Psychiatry, Autism Research Centre, University of Cambridge, 18b Trumpington Road, Autism Research Centre, Douglas House, Cambridge, CB2 8AH UK

## Abstract

**Background:**

Recent research suggests that autistic individuals have shorter lifespans and experience worse health (greater health burden) than non-autistic individuals. Small, qualitative studies suggest that autistic adults also experience poor self-reported healthcare quality.

**Methods:**

An anonymized, cross-sectional, self-report questionnaire was administered to *n* = 4158 individuals. The study assessed prevalence of chronic health conditions, healthcare quality, differences in overall health inequality score, and effects of the coronavirus pandemic on healthcare quality. We used Fisher’s exact tests, binomial logistic regression, and predictive machine learning tools, as appropriate.

**Results:**

The final sample included *n* = 2649 participants (*n* = 1285 autistic) aged 16–96 years. Autistic adults reported lower quality healthcare than non-autistic adults across 50/51 items, including poorer access to healthcare and poorer communication, alongside increased anxiety, sensory sensitivity, system-level problems, shutdowns, and meltdowns. Differences between groups were stark: aggregated health inequality scores predicted autism diagnosis, even after stratifying by sex. Autistic adults were also more likely to have chronic health conditions than non-autistic adults. There were no significant differences in healthcare quality for autistic adults before and during the pandemic, although they received relatively poorer quality healthcare than non-autistic adults across both periods.

**Limitations:**

The study’s sampling methods are not likely to capture the perspectives of all autistic individuals, especially those with intellectual disability. Both the autistic and control samples are biased towards UK residents, white individuals, those assigned female at birth, and those who completed an undergraduate degree or higher education. As such, these results may limit their generalizability to other groups. Finally, these results relate to self-reported differences in healthcare quality between autistic and non-autistic adults. The observed group differences may in part reflect differences in perception and communication rather than differences in actual healthcare quality.

**Conclusions:**

Autistic adults are more likely to have chronic health conditions alongside self-reported lower quality healthcare than others. Health inequalities between these groups are widespread and dramatic; unfortunately, they existed before and have persisted after the onset of the coronavirus pandemic.

**Supplementary Information:**

The online version contains supplementary material available at 10.1186/s13229-022-00501-w.

## Background

Autism spectrum conditions (henceforth autism) are a set of lifelong, neurodevelopmental conditions characterized by difficulties with social and communication, narrow areas of interest, and repetitive behaviors. While autism is a heterogeneous condition that may exist along the full range of intellectual ability, [1] autistic individuals may be more likely to have differences in their cognitive profile, such as atypical sensory perception, information processing, and motor abilities. Autism is common and prevalence estimates have increased in recent years, now accounting for 1 in 44 eight-year-old children today [2]. And there appears to be a sex bias in autism, with males being three to four times more likely to be diagnosed than females [2, 3]. Thus, providers from all specialties are likely to engage with autistic patients in clinical work and should be aware of the unique strengths and challenges that their autistic patients may face in regard to mental health, physical health, healthcare access, and healthcare quality.

Unfortunately, research suggests that autistic adults have an increased poorer physical and mental health [4–12], alongside increased risk of premature mortality [13–17], and greater annual health expenditure than non-autistic adults overall and across nearly all specific areas of  healthcare. These include outpatient, primary care, emergency care, mental health services, neurology, home healthcare, prescription drug claims, and skilled nursing assistance [10, 12, 18]. Unsurprisingly, diagnosis of a co-occurring physical or mental health condition further increases expenditure [10]. Autistic individuals also have higher healthcare utilization than peers, with higher likelihood of hospitalization, prescription drugs claims, and lab services, as well as a greater number of emergency room, primary care, outpatient, inpatient, mental health, neurological, and speech therapy visits [10, 12, 18, 21, 22]. However, there are a paucity of studies that describe or assess the quality of the healthcare experiences of autistic adults compared to non-autistic adults.

Quantitative, qualitative, and mixed-methods studies have considered knowledge of autism among healthcare professionals. A scoping review found that, overall, healthcare providers reported limited knowledge, resources, and training regarding autism and that they were limited in their ability to manage complex care needs of autistic patients [19]. Despite autism now being considered a relatively common condition, in at least one large-scale study healthcare professionals reported being unaware of having any adult autistic patients [20]. Healthcare professionals reported a desire for information, training, care coordination, and systemic changes to their healthcare system in order to improve the quality of their care for autistic people [19].

Only small, qualitative studies have considered the quality of healthcare experiences from the perspectives of autistic adults themselves [23–26]. Across several studies, autistic adults report difficulties in evaluating or describing their health, challenges with accessing treatment, and lack of understanding/ knowledge of autistic people across both physical and mental health services [24–27]. They also reported significant stigma regarding their autism and their need for mental health support [25, 27]. Aligning with studies of healthcare professionals, autistic adults reported that difficulties with patient-provider communication, sensory sensitivities, executive functioning, body awareness, slow processing speed, and system-level issues all served as barriers to accessing healthcare [26, 27]. A further study investigated the availability and importance of particular adjustments within UK healthcare and found that adjustments for sensory environment, knowledge and communication of the healthcare professional, and flexibility of the clinical service context (e.g., offering online appointments, changing appointment length according to patient preference, etc.) were strongly desired but infrequently available [23]. While these studies offer useful insights into challenges faced by autistic individuals, it is not clear how different these experiences are for non-autistic individuals—including others with disabilities.

Only two studies so far contrast the experiences of autistic adults and non-autistic adults about healthcare access [22, 28]. First, 209 autistic adults’ and 228 non-autistic adults’ responses to an online cross-sectional survey were evaluated using multivariate regression analyses controlling for age, sex, race/ethnicity, income, personal and parental educational attainment (as additional proxy measures of socio-economic status), health insurance, and health status [22]. They found lower satisfaction with communication, overall healthcare self-efficacy, and chronic condition self-efficacy among autistic participants compared to non-autistic peers, as well as higher odds of unmet healthcare needs for physical health, mental health, and medication [23].

Second, a community-based participatory research approach was used to consider the barriers to healthcare among 209 autistic, 55 non-autistic with disabilities, and 173 non-autistic, non-disabled individuals [28]. Interestingly, autistic participants selected different and greater barriers to health-care compared to non-autistic adults, including emotion regulation (especially fear/anxiety), patient-provider communication, slow processing speed, sensory sensitivity, concern about cost, and healthcare navigation [28]. These studies emphasize unequal access to healthcare for autistic individuals as well as additional barriers, but it is difficult to assess how generalizable these findings are due to their small sample sizes. The present study aimed to establish whether there are differences in chronic poor health and self-reported healthcare quality of autistic and non-autistic individuals across several domains (including current healthcare behavior, sensory experience, communication, anxiety, access and advocacy, system problems, and shutdowns/meltdowns). It also aimed to determine whether these issues improved or became worse after the onset of the coronavirus pandemic. While research into health and healthcare remains priorities for the autistic community [29, 30], future research may also help motivate individual providers, healthcare systems, and policymakers to consider the importance of equity in healthcare access and quality.

## Methods

### The autism and healthcare experiences survey and participant cohort

We administered an anonymous, online survey via Qualtrics on quality of healthcare, including questions regarding demographic information, a short version of the Autism Spectrum Quotient (a measure of autistic traits, AQ-10) [31, 32], current healthcare behavior, sensory experience, communication, anxiety, access and advocacy, system problems, shutdowns, meltdowns, autism-specific experiences, and most recent healthcare experience. The questionnaire included 63 questions on health and healthcare, including questions that were multiple-choice, used a 4–point Likert Scale (with the options Strongly Agree, Somewhat Agree, Somewhat Disagree, Strongly Disagree), and requested open-ended free text responses. Further information on the content of each of these sections can be found in the additional file. Specifically, Additional file 1: Figures S1-S15 provide images of each of the survey questions exactly as they were presented to participants. We used publicly available materials from the National Health Service (NHS), National Institute for Health and Care Excellence (NICE), National Institutes of Health (NIH), and the World Health Organization (WHO) to develop the survey questions. After developing a draft of the survey, we also conducted in-depth interviews (lasting several hours each) with two middle-aged autistic adults about their experiences and asked them to provide feedback on the survey study, and we revised the survey accordingly.

We employed a cross-sectional, convenience sampling design and recruited participants via the Cambridge Autism Research Database (CARD), Autistica’s Discover Network, autism charities (including the Autism Research Trust), and social media (specifically Twitter, Facebook, and Reddit). Survey collection took place from July 2019 to January 2021. Our non-autistic sample may be biased toward individuals with an interest in autism, as the study advertisements and consent form indicated that the study compares the experiences of autistic and non-autistic adults. However, we attempted to mitigate this bias by advertising our study to the general population via paid advertisements on Facebook and Reddit. In addition, we excluded all individuals who reported that they suspected they were autistic, or who were awaiting assessment, or who self-diagnosed as autistic. At all stages of recruitment, both autistic and non-autistic individuals were invited to participate. Our use of social media advertisements enabled us to attempt to recruit a diverse, international sample, including people from 79 countries.

*N* = 4158 individuals accessed the survey. Any individual aged 16 years or older who consented to participate was eligible. We excluded *n* = 1371 individuals due to incomplete response, failure to consent, or unconfirmed age (for ethical consent reasons). Although nearly all questions on the survey were optional, an incomplete response was defined as a participant who failed to complete any questions across all of the following sections: sensory experience, communication, anxiety, access and advocacy, system problems, meltdowns, shutdowns, or autism-specific experiences. In addition, a further *n* = 112 participants were excluded due to suspected duplicate response. We were able to use an existing survey setting in the Qualtrics system to prevent individuals from responding to the survey multiple times from the same IP address. However, as the survey participants were all anonymous, there was no direct way to exclude individuals who had responded to the survey multiple times. Thus, we used an algorithm to identify potential duplicate responses, excluding all participant records that matched any previous participant record across 12 criteria (autism diagnosis (yes/no), specific autism diagnosis, type of diagnosing practitioner, year of autism diagnosis, autistic family members (yes/no), age, country of residence, sex assigned at birth, current gender identity, education level, ethnicity, and AQ-10 score). Finally, as the study was anonymized, all autism diagnoses were self-reported; however, to confirm a clinical diagnosis, we asked participants to provide further information including the type of practitioner who diagnosed them (e.g., psychiatrist, clinical psychologist, pediatrician, etc.), their year of diagnosis, and their specific diagnosis (e.g., autism spectrum disorder, Asperger’s, etc.). *N* = 26 further individuals were excluded from the study (both the autistic and non-autistic cohorts), as we were unable to confirm their autism status (e.g. awaiting assessment, suspected autism, or self-diagnosed as autistic). The final sample included *n* = 2649 individuals (*n* = 1285 autistic individuals).

### Statistical analysis

We used *R Version 3.6.2* to employ unadjusted and adjusted models to assess the lifetime prevalence of mental and physical health conditions and healthcare quality across a wide variety of topics. We used Fisher’s exact tests (‘CrossTable’ function of the ‘gmodels’ package) to provide our unadjusted estimate. All adjusted analyses controlled for age, ethnicity, education level, and country of residence and employed the ‘glm’ function from the ‘stats’ package. We used a significance threshold of *p* < 0.001 for all analyses to correct for type II errors due to multiple comparisons. For adjusted analyses only, we utilized five iterations of multiple imputation for chained equations (MICE package) to address missingness across the covariates of ethnicity (5.29% missing), education level (1.13% missing), and country of residence (1.89% missing) [33]. Although nearly all questions in the survey were optional, there was very little missing data regarding outcomes (< 10% per question). Information on missing data per question has been provided in Additional file 1: Table S1. As a note, we did not impute any data for outcomes—only for covariates as specified above.

We used education level as a proxy measure of socioeconomic status. The covariate was coded as a categorical variable and was defined as the highest qualification held with the following options: 'No formal qualifications,' 'Secondary School/High School level qualifications,' 'Further vocational qualifications,' 'University Undergraduate level qualifications (BA, BSc, etc.),' and 'University Postgraduate level qualifications (MA, MSc, PhD, Certificate, etc.)'. Country of residence was also coded as a categorical variable with the following options (based on highest response frequency): 'United Kingdom,' 'United States of America,' Germany,' 'Australia,' 'Canada,' 'Netherlands' and 'Other' countries. Unfortunately, due to low response rates from individuals from all non-white ethnic backgrounds, we used a binary representation of ethnicity across all analyses. We have also included the specific details for each sub-section of analyses conducted during the study, with labeled sub-headings for ease.

### Lifetime prevalence of mental and physical health conditions

Prevalence of mental and physical health conditions may vary greatly by sex assigned at birth among individuals in the general population, as well as autistic individuals specifically [4, 5, 9, 11, 15–17]. As such, we employed sex-stratified unadjusted and adjusted analyses for individuals assigned male and female at birth to compare self-reported diagnoses of a variety of mental and physical health conditions. We have reported all findings for which an adjusted model could be fitted for both assigned female at birth and assigned male at birth individuals, although it should be noted that the results for a few conditions have not been reported due to perfect separation of the model. Perfect separation occurs when the value of a covariate directly predicts the value of the response variable (e.g. all non-white individuals are autistic); as such, the model cannot be reliably fitted, increasing the errors (and thereby 95% confidence intervals) dramatically. *N* = 4 individuals identified as 'Other' for their sex assigned at birth, but all were included in the autistic sample. Thus, these individuals were excluded from the sex-stratified analyses only.

### Healthcare experiences

We simplified the responses to the 4-point Likert scale into a binary form, in order to establish the total number of individuals who endorsed or rejected each item. This section of the questionnaire included 33 items across six categories: current healthcare behavior, sensory experience, communication, anxiety, access and advocacy, and system problems. At the top of each of these subsections, the following heading was provided: 'Please answer the following questions about your experiences of going to see a healthcare professional (Doctor, General Practitioner, Nurse Practitioner, Nurse, or Physician’s Assistant).' Images of the survey questions for these sections have been provided in Additional file 1: Figures S1–S6. Using both unadjusted and adjusted tests, we compared the frequency of endorsing each individual item between autistic and non-autistic adults. In addition, as we had self-reported information about clinical diagnoses of autism, and autistic adults may be more likely than others to be diagnosed with an anxiety disorder [4, 5, 7, 10], we also conducted a sensitivity analysis for the anxiety subsection of the survey using a binary covariate of anxiety diagnosis. There were no significant differences in effect and full results have been reported in Additional file 1: Table S4.

### Shutdowns and meltdowns

**Fig. 1:**
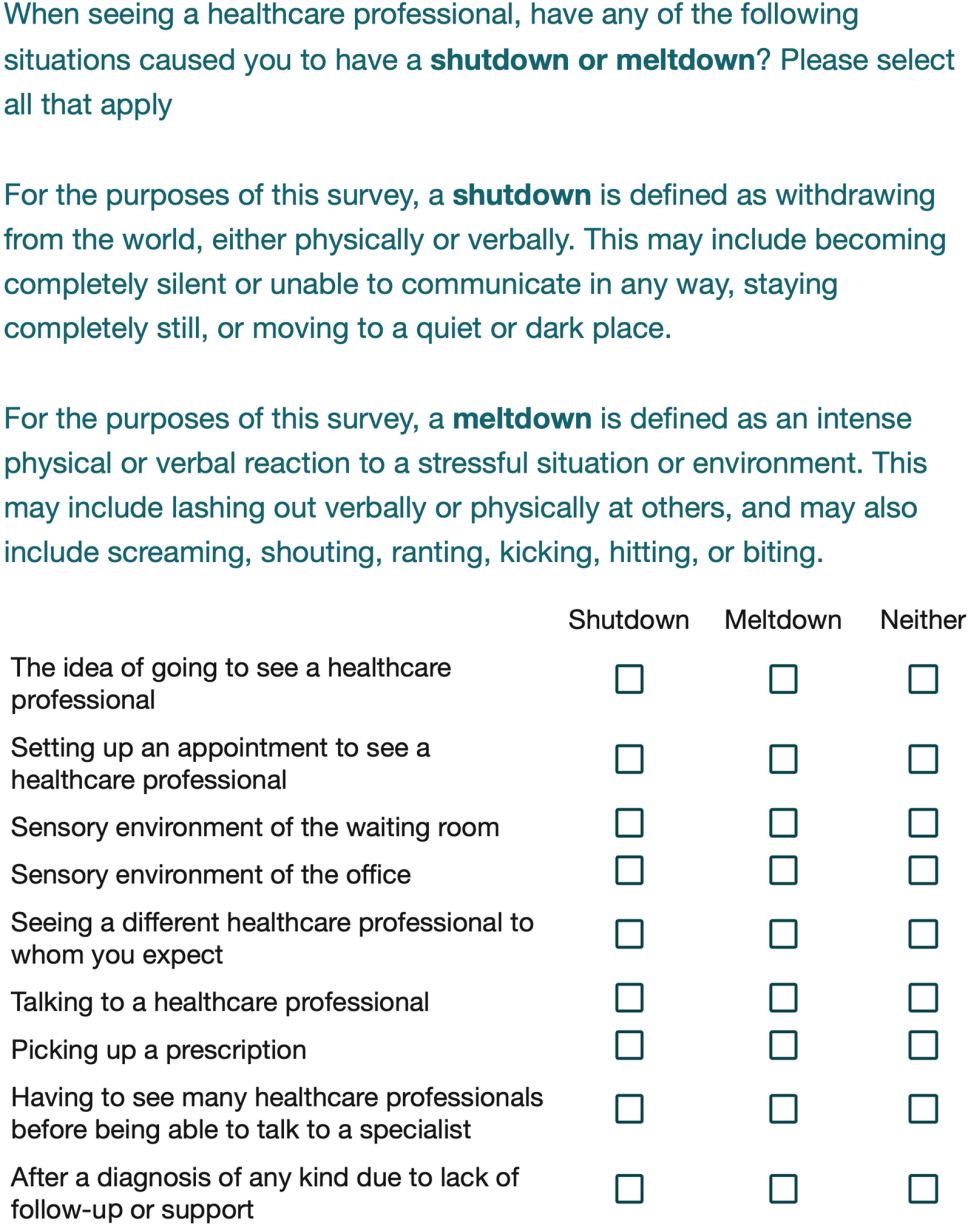
Definitions of Shutdowns and Meltdowns provided to all participants

As part of the survey, we also asked both autistic and non-autistic individuals to endorse whether a common health-related situation had ever caused a shutdown, meltdown, or neither (individuals were able to select both shutdown and meltdown for each item). These terms were defined specifically on the survey and exact phrasing can be found in Fig. 1. This section of the survey served to provide information about the severity of distress that unmet health needs or a poor-quality healthcare experience could cause autistic or non-autistic individuals. Once again, we used Fisher’s exact tests and binomial logistic regression (as described above) to determine if these experiences differed between autistic and non-autistic groups.

Figure 1 shows the question provided to all participants asking whether their previous healthcare experiences had ever resulted in shutdowns or meltdowns. All participants (both autistic and non-autistic) were also provided with the accompanying definitions of what is meant by ‘shutdown’ or ‘meltdown’ in the context of this survey.

### Healthcare inequality section scores

We then combined the individual responses on the items in the sensory experience, communication, anxiety, access and advocacy, and system problems in two different ways in order to provide a measure of overall healthcare inequality. First, we collated the items for each subsection (e.g., all of the sensory experience questions together) where each self-reported negative experience was coded as '1 point' and each positive experience was coded as '0 points.' We then added together all of the points for each subsection and divided by the total number of questions of each section to get a composite score from 0 to 1. As all items in each section were optional, only questions that were answered by each participant were factored into this calculation. However, we also ran a sensitivity analysis excluding all participants with missing data. The overall results did not change and they can be found in Additional file 1: Tables S2 and S3. Second, we used the same procedure to calculate a total score across all answered questions from any of these sections—once again, excluding unanswered questions from this analysis. Using these scores, we than ran both unadjusted and adjusted Binomial Logistic Regression models (‘glm’ function of the ‘stats’ package) to determine whether likelihood of being diagnosed with autism differed based on each of the subsection healthcare inequality scores or the total healthcare inequality score. We ran this in the whole population, as well as in sex-stratified AFAB and AMAB groups, respectively. As above, intersex participants were included in the overall analyses but not in either of the sex-stratified groups.

We tested whether we could use these healthcare inequality scores alone to correctly classify individuals as autistic or non-autistic. We used the following procedure for each subsection and the total score iteratively. First, we used the ‘createDataPartition’ function from the ‘caret’ package to section off 20% of the population (including 20% of autistic and non-autistic adults each) as a training dataset. We then used the used the ‘predict’ function from the ‘stats’ package to test whether the chosen healthcare inequality score correctly predicted autism diagnosis on the remaining 80% of the sample. The accuracy, specificity, and sensitivity of each of these tests has been reported as well. The purpose of using binomial logistic regression and machine learning methods to predict autism status based on health inequality scores was to determine whether self-reported healthcare quality was markedly different between autistic and non-autistic individuals.

### Autism specific questions

If participants self-reported an autism diagnosis at the outset of the survey, we also asked them a few additional questions about their autism-specific experiences of healthcare with their main healthcare provider (exact wording has been provided in Additional file 1: Figure S16. As these questions were not asked to the non-autistic participants, we did not conduct any statistical analyses on this section. However, we have provided the summary data for the questions in Fig. 3.

### Effects of the Covid-19 pandemic on healthcare quality

The questionnaire asked participants to answer questions about their most recent healthcare experience (screenshots of these questions have been included as Additional file 1: Figures S14 and S15). As part of this section of the survey, participants were asked to include the date of this healthcare appointment. The WHO officially designated the Covid-19 infection as a pandemic on March 11th, 2020. The present study attempted to understand whether the coronavirus pandemic has affected healthcare quality by designating healthcare appointments as ‘during the pandemic’ if they occurred on or after March 11th, 2020. Only participants who included full date information (e.g., including Date, Month, and Year of their most recent healthcare appointment) were included in these analyses. Full demographic information on this subsample can be found in Additional file 1: Table S5. We then employed Fisher’s exact tests (using the CrossTable function from the ‘gmodels’ package) to determine whether healthcare quality was different between autistic and non-autistic adults before and during the pandemic, respectively; in addition, we considered whether healthcare quality changed before and during the pandemic for autistic adults and non-autistic adults, respectively.

## Results

The sample predominantly comprised assigned female at birth and white individuals, UK residents, and those with at least a university education. There were significant group differences in mean age, ethnicity, education, and country of residence between the autistic and non-autistic groups. The mean ages were 41.26 years (sd = 14.48) and 38.40 years (sd = 16.06) for the autistic and non-autistic groups, respectively. However, there were no significant differences in sex assigned at birth between the autistic and control groups. A summary of all relevant demographic information is provided in Table 1.

**Table 1:** Participant demographics

Characteristics	Autism (*n* = 1285)	Controls (*n* = 1364)	*p*-values (Sig.)
Age (years), mean (SD)	41.26 (14.48)	38.40 (16.06)	1.44 × 10^–8^ ( ***)
Age (years), categories, N (%)			
16–29	338 (26.30)	495 (36.29)	
30–39	269 (20.93)	273 (20.01)	
40–49	274 (21.32)	238 (17.45)	
50–59	256 (19.92)	195 (14.30)	
60–69	112 (8.73)	107 (7.84)	
70 +	36 (2.80)	56 (4.11)	
AQ-10 score, mean (SD)	8.02 (1.88)	3.78 (2.60)	< 2.22 × 10^–16^ ( ***)
Female	8.13 (1.75)	3.71 (2.67)	< 2.22 × 10^–16^ ( ***)
Male	7.82 (2.07)	3.91 (2.48)	< 2.22 × 10^–16^ ( ***)
Other	8.75 (1.89)	–	
Sex assigned at birth, N (%)			0.117
Female	816 (63.50)	864 (63.34)	
Male	465 (36.19)	500 (36.66)	
Other	4 (0.31)	0	
Gender, N (%)			< 2.22 × 10^–16^ ( ***)
Female	819 (60.04)	669 (52.06)	
Male	488 (35.78)	436 (33.93)	
Non-binary	36 (2.64)	122 (9.49)	
Other^a^	21 (1.54)	56 (4.36)	
Missing	0	2 (0.16)	
Ethnicity, N (%)			9.48 × 10^–8^ ( ***)
White	1098 (85.45)	1059 (77.64)	
Non-White	179 (13.93)	299 (21.92)	
African	5 (0.39)	13 (0.95)	
Arab	1 (0.08)	8 (0.59)	
Caribbean	7 (0.54)	1 (0.07)	
Hispanic	11 (0.86)	35 (2.57)	
Jewish	25 (1.94)	36 (2.64)	
Mixed Race	88 (6.85)	125 (9.16)	
Turkish	1 (0.08)	10 (0.73)	
Other	41 (3.19)	71 (5.21)	
Missing	8 (0.62)	6 (0.44)	
Education, N (%)			2.33 × 10^–6^ ( ***)
No formal qualifications	60 (4.67)	30 (2.20)	
Further vocational qualifications	200 (15.56)	152 (11.14)	
Secondary School/ High School	220 (17.12)	250 (18.33)	
University Undergraduate	400 (31.13)	405 (29.69)	
University Postgraduate	405 (31.52)	524 (38.42)	
Missing	0	3 (0.22)	
Country of Residence			< 2.22 × 10^–16^ ( ***)
Australia	20 (1.56)	37 (2.71)	
Canada	41 (3.19)	52 (3.81)	
Germany	40 (3.11)	29 (2.13)	
Netherlands	25 (1.95)	34 (2.49)	
United Kingdom	839 (65.29)	589 (43.18)	
United States	141 (10.97)	153 (11.22)	
Other	177 (13.77)	467 (34.24)	
Missing	2 (0.16)	3 (0.22)	

*SD* standard deviation, *Sig. *significance level

*p*-values were from Pearson’s Chi Square test (categorical) or from a Mann–Whitney *U* test (means)

^a^Includes individuals who listed their sex as female and gender as male (or vice versa)

*P*-value: < .001 = * ; < .0001 = ** ; < .00001 = ***

Our sex-stratified results largely confirm previous findings regarding physical and mental health, as well as developmental conditions, suggesting that autistic individuals have increased risk of chronic conditions compared to others [4–10]. This is particularly true for autistic assigned female at birth individuals (full results shown in Table 2) compared to sex-matched peers, with the largest odds ratios being associated with mental health conditions. In regard to assigned male at birth individuals, we found that they have increased risk of mental health conditions and some developmental conditions (e.g., attention deficit hyperactivity disorder) compared to non-autistic assigned male at birth individuals, which supports previous findings [4, 6, 9]. See Table 2 for further information on comparisons between autistic and non-autistic assigned male at birth individuals for specific chronic conditions.

**Table 2:** Life time incidence of health conditions among autistic and non-autistic individuals, stratified by sex

	Unadjusted model		Adjusted model^a^		
	OR (95% CI)	*p*-value	OR (95% CI)	*p*-value	Sig
*Assigned female at birth*					
Arthritis	1.883 (1.489, 2.385)	4.78 × 10^–8^	1.864 (1.452, 2.392)	1.09 × 10^–6^	***
Breathing concern	1.617 (1.269, 2.065)	6.31 × 10^–5^	1.570 (1.228, 2.007)	3.3 × 10^–4^	*
Deafness	1.745 (1.110, 2.776)	0.013	1.850 (1.175, 2.913)	7.90 × 10^–3^	
Diabetes	1.556 (0.918, 2.670)	0.102	1.690 (0.997, 2.863)	0.051	
High blood pressure	1.098 (0.765, 1.577)	0.598	1.166 (0.804, 1.690)	0.418	
Intellectual disability	3.940 (1.744, 10.035)	3.33 × 10^–4^	3.323 (1.464, 7.541)	4.11 × 10^–3^	
Kidney/liver condition	1.522 (0.808, 2.920)	0.180	1.793 (0.961, 3.347)	0.067	
Neurological condition	2.269 (1.514, 3.449)	2.82 × 10^–5^	2.350 (1.564, 3.533)	4.11 × 10^–5^	**
Anorexia	4.238 (2.767, 6.665)	1.29 × 10^–13^	4.328 (2.805, 6.677)	4.65 × 10^–11^	***
Anxiety	4.289 (3.480, 5.296)	< 2.22 × 10^–16^	4.298 (3.454, 5.348)	< 2.22 × 10^–16^	***
ADHD	3.352 (2.346, 4.858)	7.00 × 10^–13^	3.987 (2.746, 5.789)	5.36 × 10^–13^	***
Binge eating disorder	1.391 (0.859, 2.270)	0.165	1.365 (0.847, 2.200)	0.201	
Bipolar disorder	2.641 (1.579, 4.549)	6.84 × 10^–5^	2.714 (1.612, 4.570)	1.77 × 10^–4^	*
Depression	4.097 (3.325, 5.057)	< 2.22 × 10^–16^	3.887 (3.142, 4.809)	< 2.22 × 10^–16^	***
Insomnia	2.667 (2.044, 3.496)	3.21 × 10^–14^	2.997 (2.271, 3.955)	1.42 × 10^–14^	***
Obsessive compulsive disorder	4.552 (3.035, 6.992)	2.56 × 10^–16^	4.349 (2.869, 6.592)	5.91 × 10^–12^	***
Panic disorder	2.796 (1.973, 4.008)	8.03 × 10^–10^	3.213 (2.236, 4.618)	3.52 × 10^–10^	***
Personality disorder	5.564 (3.509, 9.148)	< 2.22 × 10^–16^	5.363 (3.358, 8.564)	2.85 × 10^–12^	***
PTSD	3.232 (2.413, 4.360)	< 2.22 × 10^–16^	3.608 (2.664, 4.888)	2.22 × 10^–16^	***
Postnatal depression	1.605 (1.077, 2.411)	0.016	1.517 (1.017, 2.263)	0.041	
Schizophrenia	4.313 (1.561, 14.778)	1.89 × 10^–3^	5.162 (1.865, 14.288)	1.60 × 10^–3^	
SAD	2.741 (1.693, 4.556)	1.10 × 10^–5^	2.809 (1.728, 4.565)	3.18 × 10^–5^	**
Self-Harm	4.234 (3.220, 5.608)	< 2.22 × 10^–16^	4.405 (3.297, 5.887)	< 2.22 × 10^–16^	***
*Assigned male at birth*					
Arthritis	1.833 (1.306, 2.583)	2.95 × 10^–4^	1.451 (1.015, 2.075)	0.041	
Blindness	0.644 (0.302, 1.325)	0.241	0.705 (0.336, 1.478)	0.354	
Breathing concern	1.678 (1.221, 2.314)	1.08 × 10^–3^	1.613 (1.161, 2.241)	4.40 × 10^–3^	
Deafness	1.886 (1.152, 3.135)	9.80 × 10^–3^	1.542 (0.934, 2.545)	0.090	
Diabetes	2.187 (1.205, 4.091)	8.32 × 10^–3^	1.949 (1.059, 3.587)	0.032	
Heart condition	1.961 (1.219, 3.199)	3.42 × 10^–3^	1.944 (1.185, 3.192)	8.60 × 10^–3^	
High blood pressure	1.380 (0.995, 1.916)	0.046	1.125 (0.780, 1.623)	0.529	
Intellectual disability	3.617 (1.710, 8.347)	2.13 × 10^–4^	2.337 (1.064, 5.133)	0.034	
Kidney/liver condition	1.133 (0.585, 2.198)	0.756	1.276 (0.660, 2.466)	0.468	
Neurological condition	2.536 (1.349, 4.968)	2.43 × 10^–3^	2.670 (1.397, 5.102)	2.99 × 10^–3^	
Stroke	1.732 (0.495, 6.780)	0.408	2.010 (0.585, 6.904)	0.267	
Anorexia	1.291 (0.326, 5.386)	0.767	1.142 (0.317, 4.117)	0.839	
Anxiety	4.000 (3.008, 5.340)	< 2.22 × 10^–16^	4.042 (2.992, 5.459)	< 2.22 × 10^–16^	***
ADHD	3.227 (1.986, 5.383)	2.80 × 10^–7^	6.276 (3.645, 10.805)	5.48 × 10^–11^	***
Binge eating disorder	3.057 (1.030, 10.935)	0.035	3.345 (1.108, 10.099)	0.032	
Depression	3.318 (2.519, 4.384)	< 2.22 × 10^–16^	3.224 (2.411, 4.311)	7.11 × 10^–15^	***
Insomnia	2.903 (1.940, 4.407)	3.45 × 10^–8^	3.336 (2.184, 5.097)	3.17 × 10^–8^	***
Obsessive compulsive disorder	11.480 (4.892, 32.950)	3.67 × 10^–13^	16.834 (6.799, 41.679)	1.44 × 10^–9^	***
Panic disorder	2.824 (1.617, 5.103)	8.72 × 10^–5^	3.148 (1.771, 5.598)	9.87 × 10^–5^	***
Personality disorder	2.948 (1.409, 6.620)	1.90 × 10^–3^	3.221 (1.493, 6.947)	2.90 × 10^–3^	
PTSD	3.964 (2.231, 7.389)	1.90 × 10^–7^	4.991 (2.726, 9.137)	2.23 × 10^–7^	***
Self-harm	3.295 (1.858, 6.091)	8.60 × 10^–6^	3.855 (2.107, 7.053)	1.30 × 10^−5^	***

*OR *odds ratio, 95% *CI *95% confidence interval, *Sig. *significance level, *ADHD *attention deficit hyperactivity disorder, *SAD *seasonal affective disorder, *PTSD *post-traumatic stress disorder

^a^Binomial Logistic Regression adjusting for age, ethnicity, education, and country of residence

*P*-value: < .001 = * ; < .0001 = ** ; < .00001 = ***

Our study also investigated healthcare quality across six different sections and found overwhelmingly that autistic adults report worse quality healthcare experiences compared to non-autistic peers. There were significant differences (with autistic adults faring relatively worse compared to non-autistic adults) for all items except whether or not individuals have health insurance or are part of a national healthcare program—for which there was no significant difference. Full results are shown in Table 3. In addition, Table 3 also provides information on whether or not a common healthcare situation had triggered a meltdown or shutdown in the past. As a note, these behaviors would signal significant distress among autistic or non-autistic adults based on each common healthcare scenario. Autistic adults were 4.2–7.4 times more likely than non-autistic adults to self-report that a common healthcare scenario caused a shutdown or meltdown.

**Table 3:** Self-reported lower quality healthcare experiences for autistic adults compared to non-autistic adults

	Autistic N (%)	Non-autistic N (%)	Unadjusted model		Adjusted model^a^	
			OR (95% CI)	*p*-value	OR (95% CI)	*p*-value
Able to see healthcare professionals as often as they would like	693 (54.14)	1041 (76.38)	0.365 (0.308, 0.433)	< 2.22 × 10^–16^	0.409 (0.343, 0.487)	< 2.22 × 10^–16^
Has health insurance or is part of a national healthcare program (e.g. NHS, Medicare, Medicaid, etc.)	991 (78.78)	1095 (82.89)	0.766 (0.626, 0.937)	7.97 × 10^–3^	1.026 (0.824, 1.277)	0.818
*Sensory experience*						
Reported at least one sensory difference (hyper- or hyposensitivity)	1199 (93.31)	609 (44.65)	17.263 (13.494, 22.298)	< 2.22 × 10^–16^	17.984 (13.970, 23.152)	< 2.22 × 10^–16^
I am able to describe how my symptoms feel in my body	707 (55.58)	1174 (87.35)	0.181 (0.148, 0.221)	< 2.22 × 10^–16^	0.187 (0.152, 0.230)	< 2.22 × 10^–16^
I am able to describe how bad my pain feels	650 (51.06)	1129 (84.00)	0.199 (0.165, 0.239)	< 2.22 × 10^–16^	0.193 (0.158, 0.234)	< 2.22 × 10^–16^
I am able to describe my sensory processing differences to healthcare professionals	496 (41.96)	355 (59.97)	0.483 (0.393, 0.593)	1.00 × 10^–12^	0.480 (0.388, 0.594)	2.11 × 10^–11^
The sensory environment of the waiting room is more overwhelming than other environments	896 (70.44)	420 (31.37)	5.211 (4.398, 6.183)	< 2.22 × 10^–16^	5.253 (4.404, 6.266)	< 2.22 × 10^–16^
The sensory environment of the office is more overwhelming than other environments	745 (58.66)	343 (25.56)	4.131 (3.489, 4.896)	< 2.22 × 10^–16^	4.202 (3.524, 5.011)	< 2.22 × 10^–16^
My senses frequently overwhelm me so that I have trouble focusing on conversations with healthcare professionals	801 (62.97)	240 (17.87)	7.809 (6.503, 9.400)	< 2.22 × 10^–16^	7.587 (6.267, 9.185)	< 2.22 × 10^–16^
*Communication*						
I am usually able to explain what my symptoms are	848 (66.93)	1213 (91.27)	0.194 (0.153, 0.243)	< 2.22 × 10^–16^	0.202 (0.159, 0.256)	< 2.22 × 10^–16^
I usually understand what my healthcare professional means when they discuss my health	957 (75.47)	1251 (94.20)	0.190 (0.144, 0.248)	< 2.22 × 10^–16^	0.198 (0.150, 0.261)	< 2.22 × 10^–16^
I do not usually ask all the questions I would like to about my health	983 (77.71)	745 (56.31)	2.703 (2.271, 3.223)	< 2.22 × 10^–16^	2.503 (2.089, 2.999)	< 2.22 × 10^–16^
I can bring up a health concern even if my healthcare professional doesn't ask about it	704 (55.65)	1019 (76.96)	0.376 (0.316, 0.446)	< 2.22 × 10^–16^	0.365 (0.304, 0.439)	< 2.22 × 10^–16^
I know what is expected of me when I go to see my healthcare professional	665 (52.45)	1099 (82.82)	0.229 (0.190, 0.275)	< 2.22 × 10^–16^	0.217 (0.179, 0.264)	< 2.22 × 10^–16^
*Anxiety*						
The idea of going to see a healthcare professional makes me feel anxious	1044 (82.79)	813 (61.73)	2.981 (2.473, 3.602)	< 2.22 × 10^–16^	2.797 (2.308, 3.390)	< 2.22 × 10^–16^
The environment of the waiting room or office makes me feel anxious	1003 (79.67)	602 (45.75)	4.644 (3.887, 5.559)	< 2.22 × 10^–16^	4.509 (3.751, 5.421)	< 2.22 × 10^–16^
I feel anxious when I see a different healthcare professional to whom I expect	1053 (83.70)	595 (45.28)	6.202 (5.140, 7.505)	< 2.22 × 10^–16^	6.111 (5.036, 7.414)	< 2.22 × 10^–16^
The process of setting up an appointment makes me anxious	1053 (83.70)	715 (54.37)	4.308 (3.570, 5.212)	< 2.22 × 10^–16^	4.500 (3.700, 5.475)	< 2.22 × 10^–16^
The process of picking up a prescription makes me anxious	716 (57.05)	320 (24.37)	4.119 (3.471, 4.897)	< 2.22 × 10^–16^	4.084 (3.410, 4.892)	< 2.22 × 10^–16^
I frequently leave my healthcare professional's office feeling as though I did not receive any help at all	786 (62.43)	428 (32.55)	3.442 (2.918, 4.064)	< 2.22 × 10^–16^	3.233 (2.729, 3.830)	< 2.22 × 10^–16^
*Access and advocacy*						
Chosen not to go in to see a healthcare professional	996 (79.30)	850 (65.69)	2.000 (1.668, 2.402)	1.44 × 10^–14^	2.145 (1.777, 2.590)	3.11 × 10^–15^
I know who to contact if I have a healthcare concern	951 (75.84)	1120 (86.49)	0.491 (0.397, 0.605)	5.69 × 10^–12^	0.449 (0.359, 0.563)	4.43 × 10^–12^
If I need to go see a healthcare professional, I am able to get there	1017 (81.30)	1215 (93.82)	0.286 (0.216, 0.376)	< 2.22 × 10^–16^	0.301 (0.225, 0.401)	4.44 × 10^–16^
I usually bring someone along to help support me in my appointments	432 (34.45)	267 (20.65)	2.019 (1.684, 2.424)	6.12 × 10^–15^	2.296 (1.874, 2.813)	1.55 × 10^–15^
If I need to go to the pharmacy, I am able to get there	1101 (87.87)	1252 (96.75)	0.243 (0.167, 0.348)	< 2.22 × 10^–16^	0.294 (0.203, 0.425)	9.67 × 10^–11^
I am able to follow a procedure for next steps if asked (for example, I will attend follow-up appointments, annual checkups if applicable, etc.)	1009 (80.59)	1188 (91.88)	0.367 (0.285, 0.471)	< 2.22 × 10^–16^	0.348 (0.268, 0.452)	4.22 × 10^–15^
I am able to make appointments for myself	1022 (81.56)	1188 (92.09)	0.380 (0.294, 0.489)	2.54 × 10^–15^	0.334 (0.252, 0.443)	2.75 × 10^–14^
I will wait until it is an emergency before I go to see a healthcare professional	815 (64.99)	673 (52.09)	1.707 (1.451, 2.009)	4.02 × 10^–11^	1.619 (1.369, 1.915)	2.09 × 10^–8^
*System problems*						
In most appointments, I have enough time to discuss my concerns with healthcare professionals	515 (41.47)	880 (69.79)	0.307 (0.259, 0.363)	< 2.22 × 10^–16^	0.337 (0.284, 0.401)	< 2.22 × 10^–16^
If I need to go to see a specialist for a healthcare concern, I am able to do so	762 (61.55)	1011 (80.17)	0.396 (0.329, 0.476)	< 2.22 × 10^–16^	0.450 (0.372, 0.545)	4.44 × 10^–16^
I often choose not to go to the doctor with concerns if I need to see a specialist because I know that it will take my many appointments before I can see the specialist	702 (56.57)	494 (39.24)	2.016 (1.714, 2.373)	< 2.22 × 10^–16^	1.923 (1.625, 2.277)	4.13 × 10^–14^
I usually leave my appointments knowing what the next steps are (i.e. follow-up appointments, medications, etc.)	839 (67.66)	1052 (83.76)	0.406 (0.333, 0.493)	< 2.22 × 10^–16^	0.423 (0.345, 0.519)	2.22 × 10^–16^
I am provided with appropriate support after I receive a diagnosis of any kind (i.e. anything from infections to chronic conditions)	474 (38.23)	920 (73.25)	0.226 (0.190, 0.269)	< 2.22 × 10^–16^	0.249 (0.209, 0.297)	< 2.22 × 10^–16^
*Triggers for a shutdown*						
The idea of going to see a healthcare professional	498 (40.65)	138 (11.06)	5.506 (4.446, 6.850)	< 2.22 × 10^–16^	5.623 (4.497, 7.029)	< 2.22 × 10^–16^
Setting up an appointment to see a healthcare professional	470 (38.37)	129 (10.50)	5.305 (4.260, 6.638)	< 2.22 × 10^–16^	5.534 (4.397, 6.963)	< 2.22 × 10^–16^
Sensory environment of the waiting room	538 (43.95)	134 (10.84)	6.445 (5.196, 8.032)	< 2.22 × 10^–16^	6.249 (5.002, 7.808)	< 2.22 × 10^–16^
Sensory environment of the office	457 (37.49)	98 (7.96)	6.934 (5.453, 8.885)	< 2.22 × 10^–16^	6.659 (5.194, 8.538)	< 2.22 × 10^–16^
Seeing a different healthcare professional to whom you expect	467 (38.15)	94 (7.62)	7.475 (5.861, 9.611)	< 2.22 × 10^–16^	7.378 (5.734, 9.493)	< 2.22 × 10^–16^
Talking to a healthcare professional	504 (41.14)	130 (10.53)	5.937 (4.775, 7.419)	< 2.22 × 10^–16^	6.001 (4.777, 7.537)	< 2.22 × 10^–16^
Picking up a prescription	204 (16.68)	46 (3.74)	5.145 (3.676, 7.329)	< 2.22 × 10^–16^	5.076 (3.579, 7.201)	< 2.22 × 10^–16^
Having to see many healthcare professionals before being able to talk to a specialist	510 (41.53)	148 (11.98)	5.213 (4.229, 6.453)	< 2.22 × 10^–16^	4.949 (3.987, 6.143)	< 2.22 × 10^–16^
After a diagnosis of any kind due to lack of follow-up or support	589 (48.52)	179 (14.55)	5.529 (4.537, 6.758)	< 2.22 × 10^–16^	5.405 (4.402, 6.636)	< 2.22 × 10^–16^
*Triggers for a meltdown*						
The idea of going to see a healthcare professional	209 (17.06)	55 (4.41)	4.460 (3.257, 6.190)	< 2.22 × 10^–16^	4.372 (3.160, 6.050)	< 2.22 × 10^–16^
Setting up an appointment to see a healthcare professional	194 (15.84)	49 (3.99)	4.529 (3.255, 6.401)	< 2.22 × 10^–16^	4.212 (2.998, 5.919)	2.22 × 10^–16^
Sensory environment of the waiting room	218 (17.81)	39 (3.16)	6.648 (4.655, 9.703)	< 2.22 × 10^–16^	6.178 (4.298, 8.880)	< 2.22 × 10^–16^
Sensory environment of the office	160 (13.13)	27 (2.19)	6.738 (4.419, 10.634)	< 2.22 × 10^–16^	5.946 (3.874, 9.128)	4.44 × 10^–16^
Seeing a different healthcare professional to whom you expect	215 (17.57)	38 (3.08)	6.701 (4.676, 9.828)	< 2.22 × 10^–16^	6.078 (4.212, 8.771)	< 2.22 × 10^–16^
Talking to a healthcare professional	198 (16.16)	42 (3.40)	5.473 (3.861, 7.913)	< 2.22 × 10^–16^	5.299 (3.712, 7.565)	< 2.22 × 10^–16^
Picking up a prescription	116 (9.49)	20 (1.63)	6.330 (3.884, 10.822)	< 2.22 × 10^–16^	5.158 (3.143, 8.466)	1.01 × 10^–10^
Having to see many healthcare professionals before being able to talk to a specialist	317 (25.81)	84 (6.80)	4.765 (3.674, 6.232)	< 2.22 × 10^–16^	4.604 (3.519, 6.025)	< 2.22 × 10^–16^
After a diagnosis of any kind due to lack of follow-up or support	394 (32.46)	110 (8.94)	4.889 (3.870, 6.212)	< 2.22 × 10^–16^	4.858 (3.810, 6.195)	< 2.22 × 10^–16^

*OR *odds ratio, 95% *CI *95% confidence interval, *Sig. *significance level

^a^Binomial Logistic Regression adjusting for age, ethnicity, education, and country of residence

**Fig. 2:**
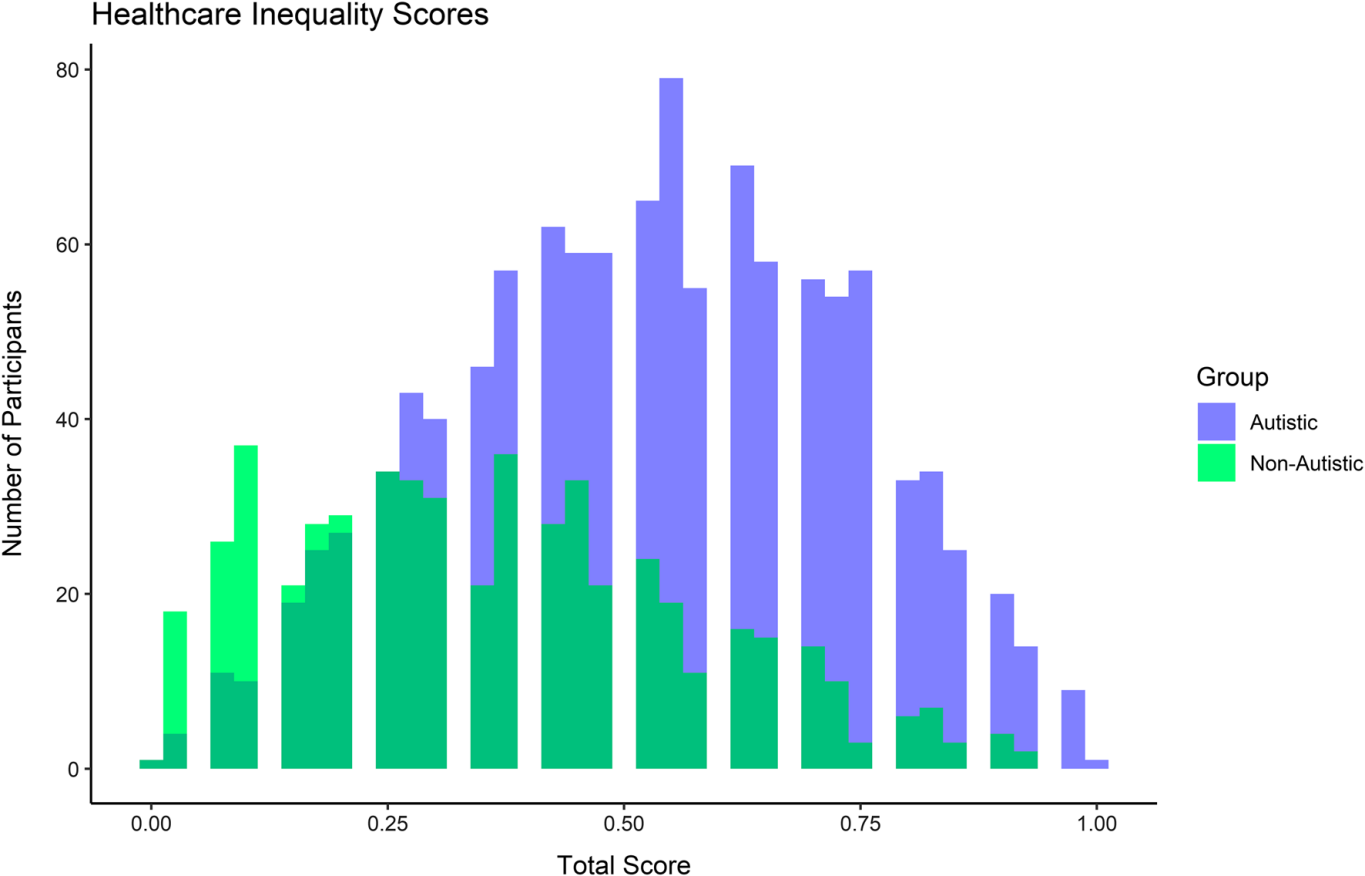
Healthcare inequality scores unadjusted distribution

We also considered trends across the aggregated scores. Results suggest that the overall score, as well as individual sub-scores for each section, are significantly different between autistic and non-autistic individuals, as well as sex stratified groups separately (e.g., autistic vs. non-autistic assigned female at birth individuals and autistic vs. non-autistic assigned male at birth individuals, respectively). Generally, across all of these subdivisions, the greatest differences were seen between the communication and anxiety subsections. Full results for the scores between autistic and non-autistic groups overall can be found in Table 4, a histogram displaying the distribution of scores between autistic and non-autistic adults can be found in Fig. 2, and Table 5 displays the sex-stratified results for the overall and subsection scores. In Table 4, the odds ratios and confidence intervals differ greatly between the unadjusted and adjusted models. The major contributing factor to the reduction in the overall point estimates for each model was the covariate of country, indicating heterogeneity in experience. Full results of these adjusted models (including estimates for each factor level of covariates) has been provided in Additional file 1: Table S6. Finally, we considered the accuracy, specificity, and sensitivity of each score in predicting autism status (in an unadjusted analysis). The results show that the overall healthcare quality score and the sensory sensitivity scores have high accuracy (> 70%) and sensitivity (> 80% or > 90%, respectively) in correctly classifying autistic and non-autistic cases alone, though they have poor specificity in doing so. Full results can be found in Table 6.

**Table 4:** Healthcare inequality scores predict likelihood of being diagnosed as autistic

	Unadjusted model^a^		Adjusted model^b^		
	OR (95% CI)	*p*-value	OR (95% CI)	*p*-value	Sig
Sensory sensitivity section score	14.687 (10.331, 21.075)	< 2.22 × 10^–16^	1.548 (1.454, 1.649)	< 2.22 × 10^–16^	***
Communication section score	18.472 (13.575, 25.330)	< 2.22 × 10^–16^	1.822 (1.702, 1.950)	< 2.22 × 10^–16^	***
Anxiety section score	22.318 (16.776, 29.912)	< 2.22 × 10^–16^	1.679 (1.596, 1.767)	< 2.22 × 10^–16^	***
Access and advocacy section score	12.328 (7.669, 20.041)	< 2.22 × 10^–16^	1.412 (1.313, 1.519)	< 2.22 × 10^–16^	***
System problems score	10.859 (8.255, 14.362)	< 2.22 × 10^–16^	1.568 (1.479, 1.662)	< 2.22 × 10^–16^	***
Total final score	41.360 (24.185, 71.825)	< 2.22 × 10^–16^	1.133 (1.110, 1.156)	< 2.22 × 10^–16^	***

*OR *odds ratio, 95% *CI *95% confidence interval, *Sig. *significance level

^a^Binomial Logistic Regression (unadjusted)

^b^Binomial Logistic Regression adjusting for age, ethnicity, education, and country of residence

*P*-value: < .001 = * ; < .0001 = ** ; < .00001 = ***

**Table 5:** Healthcare inequality scores predict likelihood of being diagnosed as autistic (sex-stratified analyses)

	Unadjusted model^a^		Adjusted model^b^		
	OR (95% CI)	*p*-value	OR (95% CI)	*p*-value	Sig.
*Assigned female at birth*					
Sensory sensitivity section score	23.548 (15.132, 37.246)	< 2.22 × 10^–16^	1.667 (1.540, 1.803)	< 2.22 × 10^–16^	***
Communication section score	30.377 (20.412, 45.838)	< 2.22 × 10^–16^	2.030 (1.859, 2.217)	< 2.22 × 10^–16^	***
Anxiety section score	45.120 (29.799, 69.518)	< 2.22 × 10^–16^	1.883 (1.750, 2.027)	< 2.22 × 10^–16^	***
Access and advocacy section score	18.205 (9.907, 34.1117)	< 2.22 × 10^–16^	1.493 (1.361, 1.638)	< 2.22 × 10^–16^	***
System problems score	16.450 (11.563, 23.634)	< 2.22 × 10^–16^	1.706 (1.583, 1.838)	< 2.22 × 10^–16^	***
Total final score	93.904 (47.026, 192.866)	< 2.22 × 10^–16^	1.165 (1.135, 1.195)	< 2.22 × 10^–16^	***
*Assigned male at birth*					
Sensory sensitivity section score	8.675 (4.613, 16.791)	5.24 × 10^–11^	1.414 (1.259, 1.587)	7.53 × 10^–9^	***
Communication section score	8.496 (5.137, 14.295)	2.34 × 10^–16^	1.527 (1.364, 1.709)	4.05 × 10^–13^	***
Anxiety section score	13.832 (8.975, 21.629)	< 2.22 × 10^–16^	1.563 (1.443, 1.693)	< 2.22 × 10^–16^	***
Access and advocacy section score	6.323 (2.969, 13.788)	2.42 × 10^–6^	1.276 (1.130, 1.441)	8.52 × 10^–5^	***
System problems score	5.831 (3.714, 9.265)	3.80 × 10^–14^	1.371 (1.244, 1.513)	3.83 × 10^–10^	***
Total final score	17.238 (6.771, 45.847)	5.09 × 10^–9^	1.096 (1.057, 1.136)	8.73 × 10^–7^	***

*OR *odds ratio, 95% *CI *95% confidence interval, *Sig. *significance level

^a^Binomial Logistic Regression (unadjusted)

^b^Binomial Logistic Regression adjusting for age, ethnicity, education, and country of residence

*P*-value: < .001 = * ; < .0001 = ** ; < .00001 = ***

**Table 6:** Healthcare inequality scores predict likelihood of being diagnosed as autistic

	Accuracy	Sensitivity	Specificity
Sensory sensitivity section score	72.100	84.501	46.868
Communication section score	65.890	61.210	70.370
Anxiety section score	70.490	62.194	78.803
Access and advocacy section score	58.020	67.275	49.177
System problems score	65.437	50.560	80.120
Total final score	72.000	93.778	25.882

Based on unadjusted binomial logistic regression analyses with autism diagnosis as the outcome variable

Figure 2 provides a visual representation of the distributions of unadjusted health inequality scores for all participants. The non-autistic participants are shown in green and the autistic participants are shown in purple. The dark green represents the overlap between the distributions of the autistic and non-autistic participants.

**Fig. 3:**
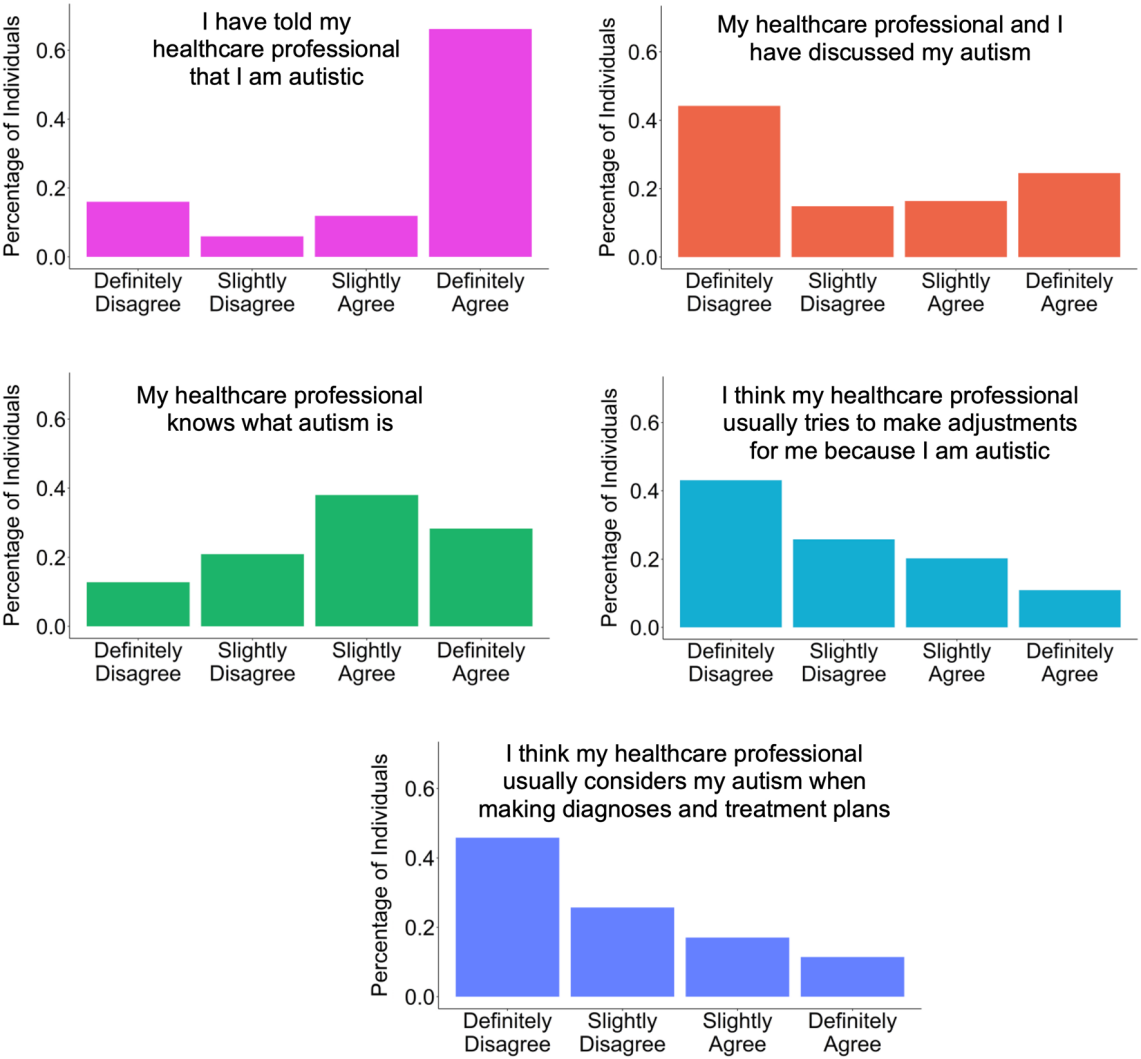
Autism-specific questions unadjusted distribution

In relation to autism specific experiences, Fig. 3 provides the unadjusted counts for the number of autistic participants who answered each question about their communication with their main healthcare professional about autism.

Figure 3 provides the unadjusted distributions of participants’ responses regarding the autism-specific healthcare questions. As this figure only shows information on autism-specific questions, only the autistic participants were provided these questions and only their responses are provided in Fig. 3.

Finally, analyzing the impact of Covid-19 on healthcare quality suggested that autistic adults experienced poorer quality healthcare overall than non-autistic adults both before the onset of the coronavirus pandemic and thereafter. However, there was no significant difference between the proportion of autistic or non-autistic adults that endorsed the statement '*I do not think that the healthcare professional cared about my wellbeing*' before or during the pandemic. Full results are provided in Table 7 and full demographic details about the sample used in the analyses related to Covid-19 are provided in Additional file 1: Table S5. There were no significant differences in healthcare quality comparing healthcare experiences before and after the onset of the pandemic among autistic adults or non-autistic adults that met our significance threshold of *p* < 0.001. Results from that analysis are provided in Additional file 1: Table S6.

**Table 7:** Autistic adults report lower quality healthcare than non-autistic peers both before and during the pandemic

	Autistic group *N* (%)	Control group *N* (%)	Odds ratio (95% CI)	*p*-value	Sig.
*Pre-Pandemic*					
I understood the questions my healthcare professional asked	777 (88.60)	700 (96.82)	0.256 (0.153, 0.411)	2.04 × 10^–10^	***
The healthcare professional gave me enough time	630 (71.84)	617 (85.34)	0.438 (0.337, 0.568)	6.97 × 10^–11^	***
The healthcare professional understood me when I described my symptoms	616 (70.24)	628 (86.86)	0.357 (0.272, 0.466)	8.39 × 10^–16^	***
The healthcare professional attempted to help me with my symptoms	675 (76.97)	629 (87.00)	0.500 (0.378, 0.657)	2.10 × 10^–7^	***
I do not think that the healthcare professional cared about my wellbeing	224 (25.54)	135 (18.67)	1.494 (1.167, 1.917)	0.001	
*During Pandemic*					
I understood the questions my healthcare professional asked	242 (88.97)	401 (97.09)	0.242 (0.111, 0.498)	2.66 × 10^–5^	***
The healthcare professional gave me enough time	200 (73.53)	363 (87.89)	0.383 (0.251, 0.582)	2.23 × 10^–6^	***
The healthcare professional understood me when I described my symptoms	196 (72.06)	369 (89.35)	0.308 (0.199, 0.472)	9.96 × 10^–9^	***
The healthcare professional attempted to help me with my symptoms	211 (77.57)	377 (91.28)	0.331 (0.205, 0.527)	9.87 × 10^–7^	***
I do not think that the healthcare professional cared about my wellbeing	54 (19.85)	79 (19.13)	1.047 (0.697, 1.566)	0.844	

95% *CI *95% confidence interval, *Sig. *Significance level

*P*-value: < .001 = * ; < .0001 = ** ; < .00001 = ***

## Discussion

Differences in self-reported healthcare quality between autistic and non-autistic adults are dramatic, with autistic adults reporting lower quality healthcare experiences across 50/51 items. In addition to affecting healthcare quality itself, these experiences are also contributing to far greater rates of anxiety, shutdowns, and meltdowns associated with common healthcare experiences than among non-autistic people. Further, this is the first study to quantify that these issues exist individually and are also clear when aggregated into a health inequality score (overall and for each of the sub-domains). The results replicate findings from earlier cross-sectional studies of medical databases and self-report surveys to suggest that autistic assigned female and assigned male at birth individuals have increased risks of mental and/or physical health conditions compared to sex-matched peers [4, 12]. This includes 1.6–5.4 times increased risk of arthritis, breathing concerns, neurological conditions, anorexia, bipolar disorder, personality disorder, and seasonal affective disorder (SAD) among autistic compared to non-autistic assigned female at birth individuals, as well as 3.0–16.8 times increased risk of anxiety, attention deficit hyperactivity disorder (ADHD), depression, insomnia, obsessive compulsive disorder (OCD), panic disorder, post-traumatic stress disorder (PTSD), and self-harm among autistic vs. non-autistic sex-matched groups. When using a *p*-threshold of < 0.001, there were no significant differences in risk of physical health conditions between autistic and non-autistic assigned male at birth groups. However, previous studies suggest otherwise [4, 7, 11], and the study supports increased risk of arthritis, breathing concerns, diabetes, heart conditions, and neurological conditions among autistic compared to non-autistic assigned male at birth individuals when using a less stringent p-threshold of 0.05. The sample is heavily biased toward assigned female at birth individuals (comprising approximately 63% of both the autistic and non-autistic groups). This was expected based on the study methodology, as they are more likely to participate in online, self-report survey studies [34–37]. However, taken together, these factors suggest that the lack of significant differences in lifetime prevalence of physical health conditions between assigned male at birth groups may be due to underpowered analyses.

Previous studies support increased healthcare expenditure, and the current study confirms no difference in access to health insurance or national healthcare program between autistic or non-autistic adults [10, 12, 18]. However, the analyses also suggest that for every 10 non-autistic adults that report that they are able to see a healthcare professional as often as they would like, only four autistic adults report the same (OR: 0.409; 95% CI: 0.343, 0.487, *p*-value: < 0.001). These findings emphasize differences in demand and perceived access to healthcare between autistic and non-autistic adults, despite some indicators of similar levels of basic healthcare coverage. This discrepancy may be accounted for by complex care needs accompanied by systemic issues, noted previously by both providers and autistic adults themselves [19, 27]. In addition, it may be a result of sweeping differences in self-reported healthcare quality between autistic and non-autistic adults, overall and in the areas of sensory experience, communication, anxiety, access and advocacy, and system problems. Notably, these differences are so stark that the derived healthcare quality score can predict autism diagnosis with 72% accuracy and 94% sensitivity in unadjusted regression analyses.

The results of the present study suggest that differences in healthcare quality between autistic and non-autistic adults existed before and persisted after the onset of the coronavirus pandemic, which clarifies that the stark contrasts in healthcare quality preceded the onset of the coronavirus pandemic. There were no significant differences in healthcare quality before and during the pandemic among autistic adults. This may be representative of the consistency of healthcare quality for autistic adults over time—or these analyses may have been underpowered, as this sub-sample included a relatively smaller proportion of participants whose most recent healthcare appointment occurred after the onset of the pandemic. When using a less stringent *p*-threshold of 0.05, non-autistic adults’ healthcare improved after the onset of the pandemic across at least one measure. Specifically, non-autistic adults were more likely to endorse the statement ''*The healthcare professional attempted to help me with my symptoms'* after the onset of the pandemic than they were before. All other measures of healthcare quality were not significantly different for non-autistic adults before and after the onset of the pandemic.

One of the defining features of autism is differences in social communication, and the study supports that these differences may affect perceived healthcare quality. Autistic adults are far less likely than non-autistic adults (specifically, 20–36% as likely) to report being able to describe their symptoms, understand what their healthcare professional means, or bring up a healthcare concern if not prompted by a healthcare professional. They are also over twice as likely as non-autistic adults to report not asking all the questions they would like to when meeting with a healthcare professional. While the study focuses on perceived healthcare communication from the perspective of the patient, it has also been suggested that differences in social communication may contribute to two-way communication difficulties between autistic and non-autistic individuals (perhaps related to the ‘double empathy’ problem) [38, 39]. There is some evidence that these challenges may apply in healthcare settings, as physicians have previously noted that challenges with communication with patients as well as caregivers can serve as barriers to care [19]. The study is not able to test directly whether this phenomenon could apply in a healthcare context, as we did not collect data from healthcare providers as part of this study. Future research should consider perceived communication between patients and providers from both perspectives, in order to understand these dynamics more clearly and develop appropriate strategies for both parties to improve communication.

Sensory sensitivities are now becoming better recognized as a core feature of the autistic experience and have been mentioned in the most recent guidance from the DSM, ICD, and NICE (DSM-5, 2013; ICD-11, 2020; NICE, 2016). Their relevance to healthcare has been recognized previously in studies of healthcare professionals and qualitative accounts from autistic adults [19, 23, 26, 27]. However, their role in affecting healthcare quality has not yet been investigated in large, comparative samples of autistic and non-autistic adults. Strikingly, for every 10 non-autistic participants that reported being able to describe how their symptoms, pain, or sensory sensitivities feel in their body to healthcare professionals, only 2–4 autistic participants reported the same. Autistic adults were also over four times more likely than non-autistic adults to report sensory overload related to the healthcare environment, and over seven times more likely to report that frequent sensory overload made it difficult to focus on conversations with healthcare professionals.

The results also indicate that these differences in healthcare quality relate to more severe disruption for autistic than non-autistic adults, characterized by increased anxiety, as well as increased likelihood of experiencing shutdowns and meltdowns related to common healthcare situations. These differences not only affect the quality of life and healthcare experiences of autistic adults, but also may relate to access as well. Autistic adults were twice as likely as others to report choosing not to go see a healthcare professional, 1.6 times as likely to endorse that they would '…wait until it is an emergency before I go to see a healthcare professional,' and over three times more likely to report frequently leaving healthcare appointments feeling as if they have not had any help at all.

Difficulties with healthcare professionals appear to be presenting at multiple levels, both in understanding expectations and next steps (in the context of follow-up appointments, medications, specialist referrals, etc.), as well as completing necessary steps/ advocating for oneself even in the case of known expectations, possibly due to systemic barriers. These results have significant implications for the improvement of healthcare of autistic adults, as they provide early indication that additional support must have the dual focus of improving the quality of communication—particularly in regard to provider expectations of their patients—as well as ensuring that autistic patients and/ or their advocates are able to carry out necessary tasks. Future research should focus on identifying the underlying causes of these difficulties; however, one possible intervention may be lengthening patient appointments—as the study replicates previous findings to suggest that autistic adults are more likely than non-autistic peers to report not having enough time in healthcare appointments [22, 23].

### Limitations

While this is the largest study of healthcare experiences of autistic adults, as well as the first large-scale study comparing their experiences to those of non-autistic adults, there are several limitations that should be kept in mind. First, these results relate to perceived differences in healthcare quality among autistic and non-autistic adults—rather than information about the number of appointments or average expenditure. Differences in social communication are a core feature of autism. As such, autistic individuals may be more candid about their experiences than others, which may partly explain differences in perceived healthcare quality. Second, the results of this study are not generalizable to the entire autistic population. Due to the use of a lengthy, online, self-report survey, the study is unlikely to represent the experiences of autistic individuals with moderate to severe intellectual disability, those without internet access, or those that are not able to fill in a lengthy online survey. Third, the sample was biased toward assigned female at birth individuals, UK residents, white individuals, and those who had completed an undergraduate degree or higher education—limiting its applicability to other groups. Fourth, the study employed a cross-sectional, convenience-sampling design and the recruitment via autism databases and social media likely left the study open to sampling bias. Therefore, it is possible that these biases may have resulted in under- or over-estimations of true group differences in prevalence of health conditions or healthcare quality. Fifth, while the study attempted to recruit adults from the general population for the study as a comparison group (particularly using advertisements without initial mentions of autism on Facebook and Reddit), the non-autistic group may be biased toward individuals with an interest in autism, undiagnosed autism, or those who suspect that they may have autism. To mitigate this risk, all individuals were excluded (from both the autistic and non-autistic groups) who reported self-diagnosing autism, suspected autism, or were awaiting autism diagnosis. In addition, the mean AQ-10 scores and Wilcoxon signed rank test results reported in Table 7 show clear delineation between autistic and non-autistic groups (as well as between autistic vs. non-autistic assigned female at birth individuals and autistic vs. non-autistic assigned male at birth individuals, respectively), in regard to autistic traits. Still, it is possible that the results may underestimate true group differences between autistic and non-autistic adults regarding lifetime prevalence of health conditions or healthcare quality. Finally, the study may be subject to winner’s curse, a phenomena common to epidemiology and genetics in which odds ratios are artificially inflated for the first study reporting a significant difference than later studies of the same group; as such, future research should work to confirm these findings and effect sizes.

As noted for several reasons above, the point estimates and effect sizes reported above may not be representative of true risk between autistic and non-autistic adults, underlining the importance of future studies that employ a variety of methodologies. However, the present study shows the widespread group differences between autistic and non-autistic adults in self-reported quality of healthcare.

## Conclusions

These results should be of significant concern to individual providers, as well as larger healthcare organizations, as the present study overwhelmingly suggests that healthcare access and quality for autistic adults is not equitable to that of their non-autistic peers. This is very concerning in light of evidence of the increased risks of mental and physical health problems, as well as premature mortality among autistic individuals [4–17]. Clinicians, policymakers, and researchers must work cooperatively with autistic individuals and their advocates to identify strategies for improving patient-provider communication, reducing sensory distress and anxiety in appointments, and minimizing system-level barriers to access. This may include improving and expanding health education on autism for healthcare professionals, as well as evaluating the efficacy of structural changes in available reasonable adjustments for autistic adults in healthcare settings (e.g., lengthened appointments, alternative forms of communication/appointments, annual healthcare maintenance checks, etc.).

Autism is now widely considered to be a relatively common condition, comprising about 2% of eight-year-old children today—and rates of autism diagnosis have continued to increase in recent years [2]. As such, healthcare systems are not only failing to provide appropriate care to autistic adults, but also that these failures may be detrimentally affecting the length and quality of life of autistic individuals. Future research should focus on determining whether healthcare quality directly relates to poorer health outcomes. It is urgent that individual healthcare professionals, healthcare organizations, and policymakers work cooperatively with autistic patients and advocates to develop and implement strategies that improve healthcare quality and access.

## Supplementary Information

**Additional file 1.** The additional file includes images of the survey (as shown to participants), information on missing data, information on sensitivity analyses, and further analyses showing non-significant differences in healthcare quality related the Covid-19 pandemic.

## Data Availability

We can provide group level data but not the underlying material itself, as our participants did not consent to having their data shared publicly. Underlying, anonymized data will be stored until 2026 and will only be made available to potential collaborators with ethical approval, after they submit a research proposal to the Autism Research Centre, University of Cambridge, UK, as is required by our original ethics application and participant consent form.
