# Supplementary material for: Autistic adults have poorer quality healthcare and worse health based on self-report data
Source: Mol Autism. 2022 May 26;13:23. doi: 10.1186/s13229-022-00501-w (PMC9135388; doi:10.1186/s13229-022-00501-w)
Supplement: Supplementary file 1 — Additional file 1. The additional file includes images of the survey (as shown to participants), information on missing data, information on sensitivity analyses, and further analyses showing non-significant differences in healthcare quality related the Covid-19 pandemic. [file 13229_2022_501_MOESM1_ESM.pdf]

Supplementary Material

Supplementary Figure S1: Part Two Heading....2
Supplementary Figure S2: Current Healthcare Behavior....3
Supplementary Figure S3: Health Insurance....4
Supplementary Figure S4: Chronic Physical Health Conditions....5
Supplementary Figure S5: Chronic Mental Health Conditions....6
Supplementary Figure S6: Differences in Sensory Processing....7
Supplementary Figure S7: Sensory Experiences of Healthcare....8
Supplementary Figure S8: Communication during Healthcare Experiences....9
Supplementary Figure S9: Anxiety from Healthcare Experiences....10
Supplementary Figure S10: Access and Advocacy Related to Healthcare....11
Supplementary Figure S11: Choice not to Seek Help for Health Concern....12
Supplementary Figure S12: System Problems Regarding Healthcare....13
Supplementary Figure S13: Part Three Heading....14
Supplementary Figure S14: Demographic Details about Most Recent Healthcare Experience....15
Supplementary Figure S15: Content of Most Recent Healthcare Appointment....16
Supplementary Figure 16: Autism-Specific Healthcare From Main Healthcare Professional....17
Supplementary Table S1: Missing Data For Outcomes....18
Sensitivity Analysis Information 1: Healthcare Inequality Scores....20
Supplementary Table S2: Sensitivity Analysis of Healthcare Inequality Scores with Complete Cases Only....21
Supplementary Table S3: Sensitivity Analysis of Healthcare Inequality Scores with Complete Cases Only....22
Sensitivity Analysis Information 2: Individuals Diagnosed with Anxiety....22
Supplementary Table S4: Sensitivity Analysis for Individuals with Anxiety....23
Supplementary Table S5: Participant Demographics for Covid Analyses....24
Supplementary Table S6: No Significant Differences in Healthcare Quality for Autistic or Non-autistic Adults Before and After the Onset of the Pandemic....25
Supplementary Table S7: Full Results for Binomial Logistic Regression Predicting Autism Status from Health Inequality Scores....26

Supplementary Figure S1: Part Two Heading

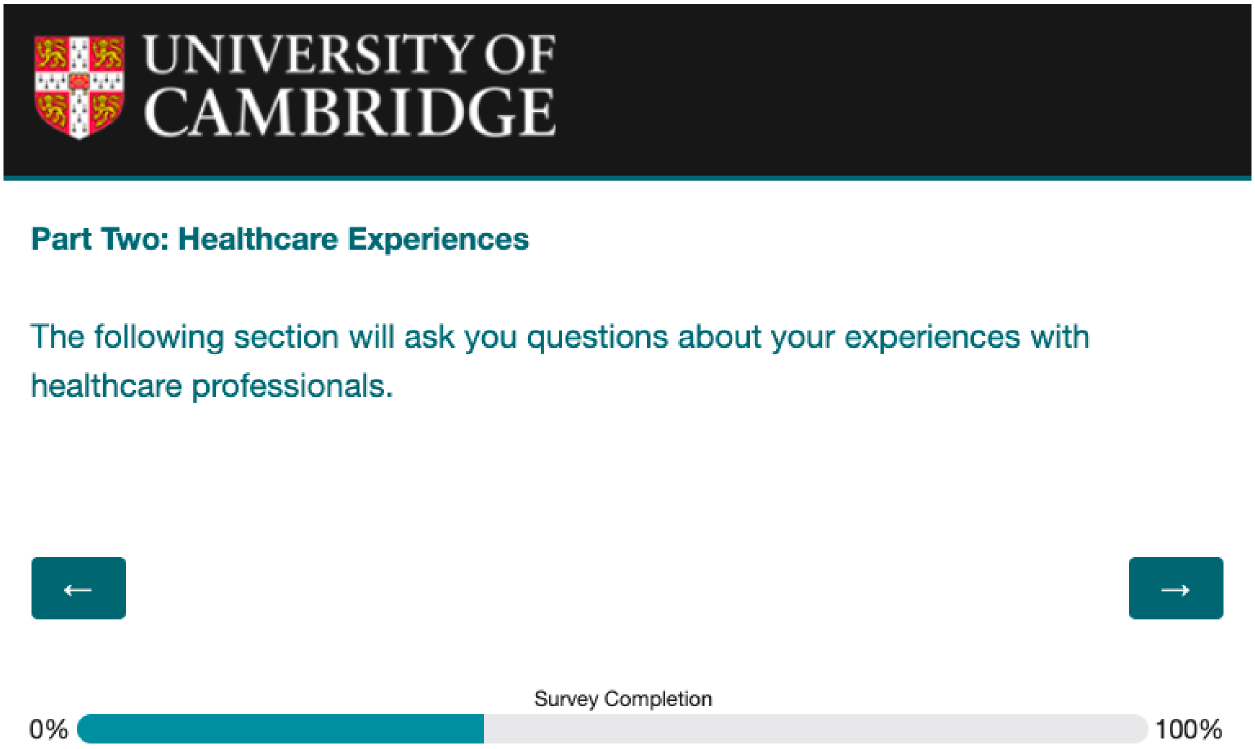

Supplementary Figure S2: Current Healthcare Behavior

For the purposes of this survey, healthcare professionals will include Doctors, General Practitioners, Nurse Practitioners, Nurses, and Physician's Assistants. Please answer the following questions when considering your interactions with these kinds of healthcare professionals.

How often do you go to see a healthcare professional regarding your own healthcare (i.e. not about a child, parent, partner, etc...)? Please select the option that most closely applies.

- ◯ Daily
- ◯ 2-3 times per week
- ◯ Weekly
- ◯ Monthly
- ◯ Every 6 months
- ◯ Yearly
- ◯ Only in emergencies
- ◯ Other: ________

Are you able to see healthcare professionals as often as you would like?

- ◯ Yes
- ◯ No

When you go in to see a healthcare professional, are you able to see the same person each time? Please select the option that most closely applies.

- ◯ I am usually able to see the same person
- ◯ I would like to see the same person each time, but usually have to see different people
- ◯ I do not have a preference about who I see
- ◯ I only seek healthcare if there is an emergency
- ◯ Other: ________

Supplementary Figure S3: Health Insurance

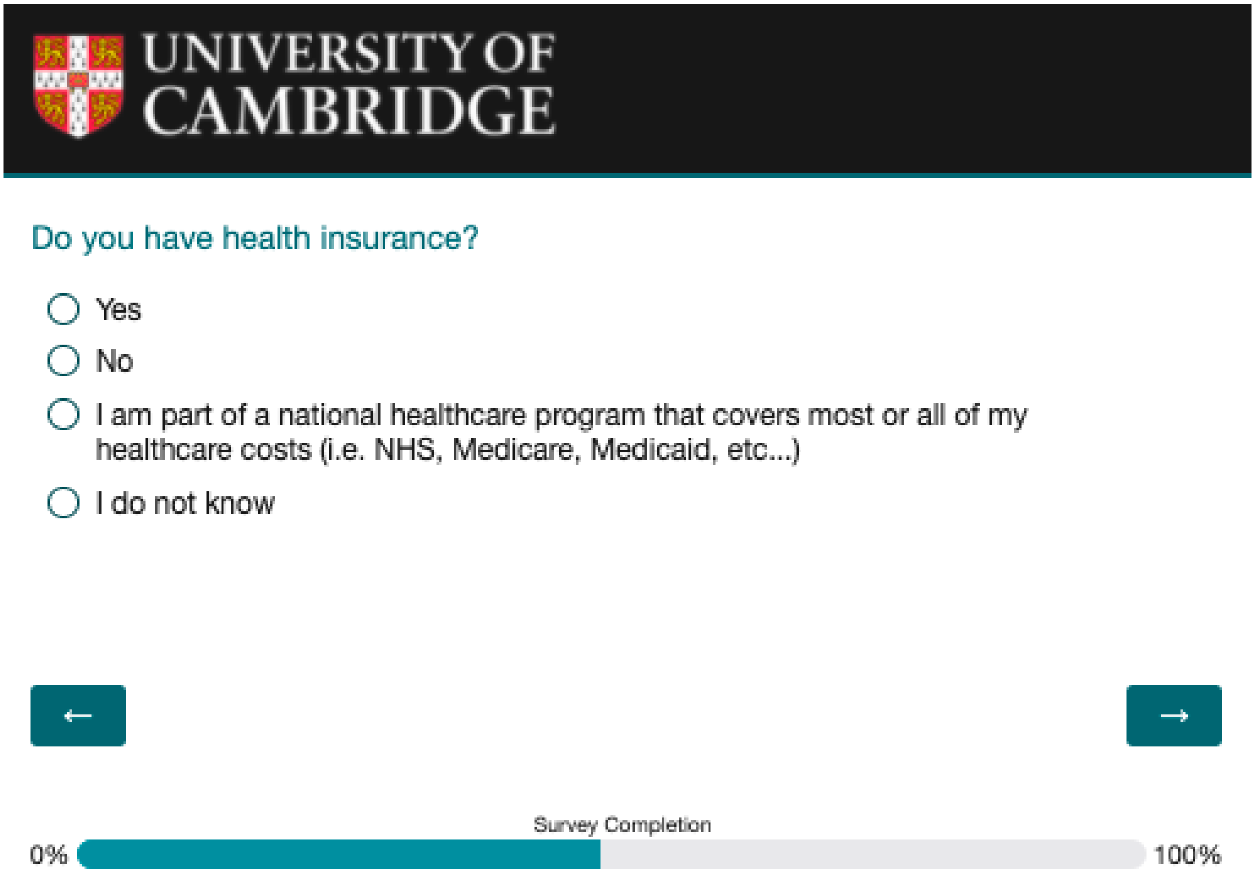

UNIVERSITY OF CAMBRIDGE

Do you have health insurance?

- Yes
- No
- I am part of a national healthcare program that covers most or all of my healthcare costs (i.e. NHS, Medicare, Medicaid, etc...)
- I do not know

← →

Survey Completion

0% 100%

Supplementary Figure S4: Chronic Physical Health Conditions

Have you ever been diagnosed with any of the following physical health conditions by a healthcare professional (Doctor, General Practitioner, Nurse Practitioner, Nurse, or Physician's Assistant)?

	Yes	No, but a healthcare professional recommended assessment	No, I suspect that I have this condition but have not been offered assessment	No
Dementia (Including Alzheimer's Disease)				
Arthritis/ Ongoing Problems with Back or Joints				
Blindness/ Partial Sight				
Breathing Condition (Asthma, COPD, etc)				
Cancer				
Deafness or Hearing Loss				
Diabetes				
Heart Condition (Angina, Atrial Fibrillation, etc)				
High Blood Pressure				
Intellectual Disability				
Kidney or Liver Disease				
Neurological Condition (Epilepsy, Multiple Sclerosis, etc)				
Stroke				
Other:				

Supplementary Figure S5: Chronic Mental Health Conditions

Have you been diagnosed with any of the following mental health conditions by a healthcare professional (Doctor, General Practitioner, Nurse Practitioner, Nurse, or Physician's Assistant)?

	Yes	No, but a healthcare professional recommended assessment	No, I suspect that I have this condition but have not been offered assessment	No
Anorexia Nervosa	○	○	○	○
Anxiety	○	○	○	○
Attention Deficit Hyperactivity Disorder (ADHD)	○	○	○	○
Binge Eating	○	○	○	○
Bipolar Disorder	○	○	○	○
Bulimia	○	○	○	○
Depression	○	○	○	○
Insomnia	○	○	○	○
Obsessive Compulsive Disorder (OCD)	○	○	○	○
Panic Disorder	○	○	○	○
Personality Disorder	○	○	○	○
Post-Traumatic Stress Disorder (PTSD)	○	○	○	○
Postnatal Depression	○	○	○	○
Schizophrenia	○	○	○	○
Seasonal Affective Disorder (SAD)	○	○	○	○
Self-Harm	○	○	○	○
Other: ______	○	○	○	○

Supplementary Figure S6: Differences in Sensory Processing

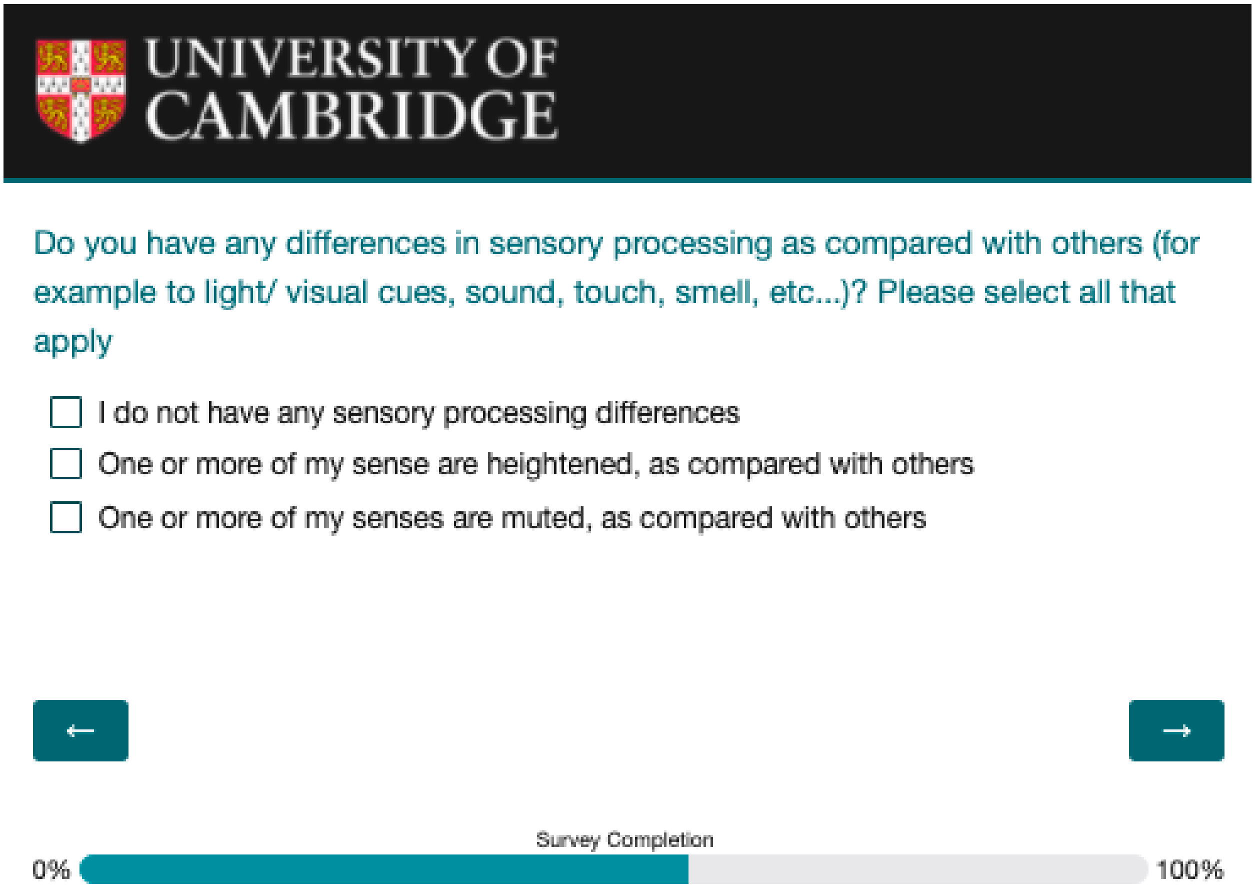

UNIVERSITY OF CAMBRIDGE

Do you have any differences in sensory processing as compared with others (for example to light/ visual cues, sound, touch, smell, etc...)? Please select all that apply

- [ ] I do not have any sensory processing differences
- [ ] One or more of my sense are heightened, as compared with others
- [ ] One or more of my senses are muted, as compared with others

← →

Survey Completion

0% 100%

Supplementary Figure S7: Sensory Experiences of Healthcare

UNIVERSITY OF CAMBRIDGE

Please answer the following questions about your experiences of going to see a healthcare professional (Doctor, General Practitioner, Nurse Practitioner, Nurse, or Physician's Assistant)

	Definitely Agree	Slightly Agree	Slightly Disagree	Definitely Disagree
I am able to describe how my symptoms feel in my body	○	○	○	○
I am able to describe how bad my pain feels	○	○	○	○
I am able to describe my sensory processing differences to healthcare professionals	○	○	○	○
The sensory environment of the waiting room is more overwhelming than other environments	○	○	○	○
The sensory environment of the office is more overwhelming than other environments	○	○	○	○
My senses frequently overwhelm me so that I have trouble focusing on conversations with healthcare professionals	○	○	○	○

Supplementary Figure S8: Communication during Healthcare Experiences

UNIVERSITY OF CAMBRIDGE

Please answer the following questions about your experiences of going to see a healthcare professional (Doctor, General Practitioner, Nurse Practitioner, Nurse, or Physician's Assistant)

	Definitely Agree	Slightly Agree	Slightly Disagree	Definitely Disagree
I am usually able to explain what my symptoms are	○	○	○	○
I usually understand what my healthcare provider means when they discuss my health	○	○	○	○
I do not usually ask all the questions I would like to about my health	○	○	○	○
I can bring up a health concern even if my healthcare professional doesn't ask about it	○	○	○	○
I know what is expected of me when I go to see my healthcare professional	○	○	○	○

Supplementary Figure S9: Anxiety from Healthcare Experiences

UNIVERSITY OF CAMBRIDGE

Please answer the following questions about your experiences of going to see a healthcare professional (Doctor, General Practitioner, Nurse Practitioner, Nurse, or Physician's Assistant)

	Definitely Agree	Slightly Agree	Slightly Disagree	Definitely Disagree
The idea of going to see a healthcare professional makes me feel anxious				
The environment of the waiting room or office makes me feel anxious				
I feel anxious when I see a different healthcare professional to whom I expect				
The process of setting up an appointment makes me anxious				
The process of picking up a prescription makes me anxious				
I frequently leave my healthcare professional's office feeling as though I did not receive any help at all				

Supplementary Figure S10: Access and Advocacy Related to Healthcare

Please answer the following questions about your experiences of going to see a healthcare professional (Doctor, General Practitioner, Nurse Practitioner, Nurse, or Physician's Assistant)

	Definitely Agree	Slightly Agree	Slightly Disagree	Definitely Disagree
I know who to contact if I have a healthcare concern	○	○	○	○
If I need to go to see a healthcare professional, I am able to get there	○	○	○	○
I usually bring someone along to help support me in my appointments	○	○	○	○
If I need to go to the pharmacy, I am able to get there	○	○	○	○
I able to follow a procedure for next steps if asked (for example, I will attend follow-up appointments, annual checkups if applicable, etc...)	○	○	○	○
I am able to make appointments for myself	○	○	○	○
I will wait until it is an emergency before I go to see a healthcare professional	○	○	○	○

Supplementary Figure S11: Choice not to Seek Help for Health Concern

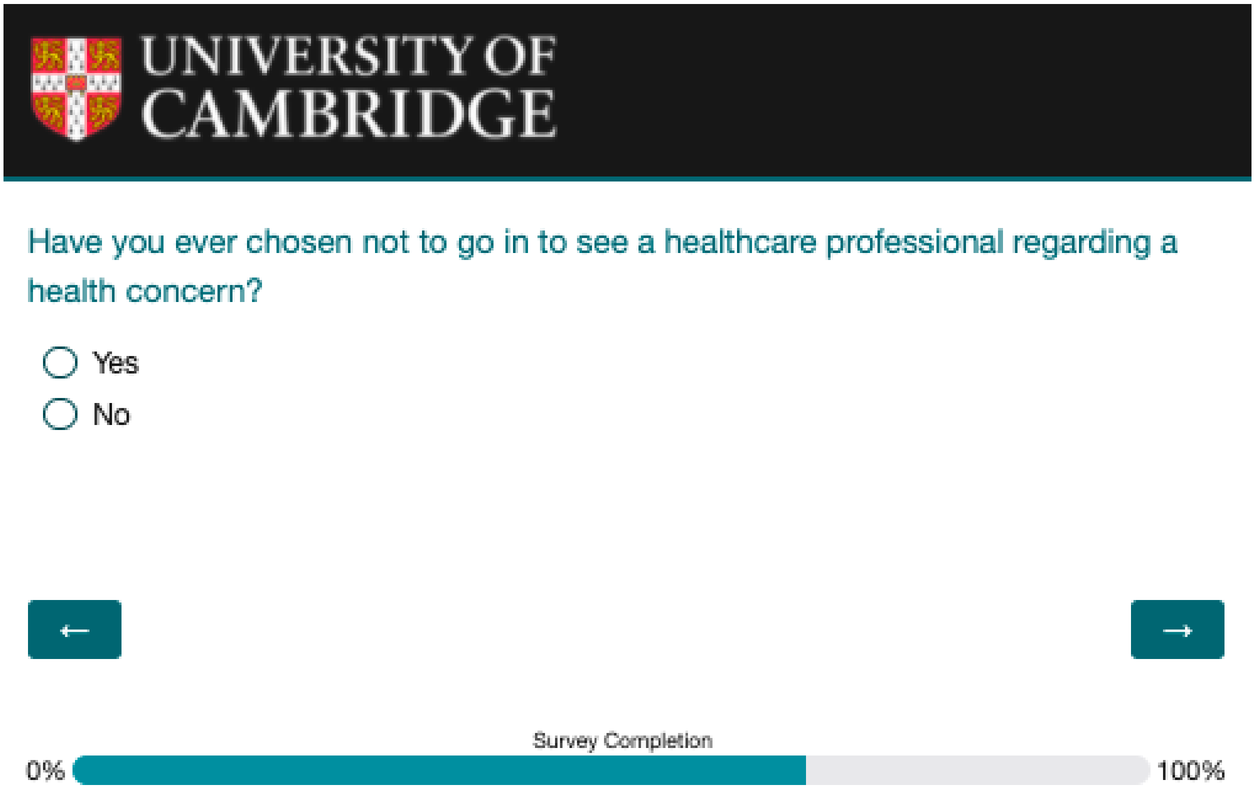
UNIVERSITY OF CAMBRIDGE

Have you ever chosen not to go in to see a healthcare professional regarding a health concern?

- Yes
- No

← →

Survey Completion

0% 100%

Supplementary Figure S12: System Problems Regarding Healthcare

Please answer the following questions about your experiences of going to see a healthcare professional (Doctor, General Practitioner, Nurse Practitioner, Nurse, or Physician's Assistant)

	Definitely Agree	Slightly Agree	Slightly Disagree	Definitely Disagree
In most appointments, I have enough time to discuss my concerns with healthcare professionals	○	○	○	○
If I need to go to see a specialist for a healthcare concern, I am able to do so	○	○	○	○
I often choose not to go to the doctor with concerns if I need to see a specialist because I know that it will take me many appointments before I can see the specialist	○	○	○	○
I usually leave my appointments knowing what the next steps are (ie follow-up appointments, medications, etc)	○	○	○	○
I am provided with appropriate support after I receive a diagnosis of any kind (ie anything from infections to chronic conditions)	○	○	○	○

Supplementary Figure S13: Part Three Heading

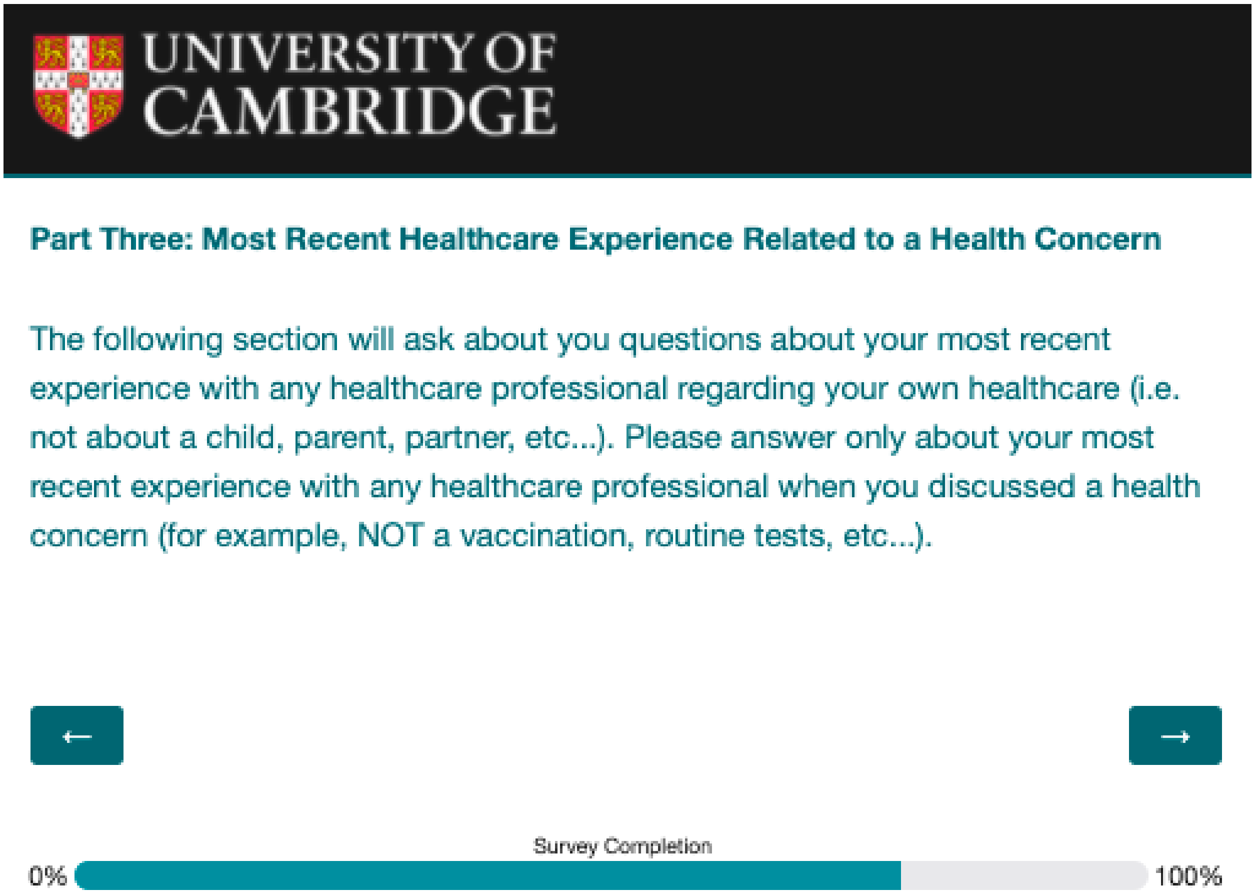
UNIVERSITY OF CAMBRIDGE

Part Three: Most Recent Healthcare Experience Related to a Health Concern

The following section will ask about you questions about your most recent experience with any healthcare professional regarding your own healthcare (i.e. not about a child, parent, partner, etc...). Please answer only about your most recent experience with any healthcare professional when you discussed a health concern (for example, NOT a vaccination, routine tests, etc...).

← →

Survey Completion

0% 100%

Supplementary Figure S14: Demographic Details about Most Recent Healthcare Experience

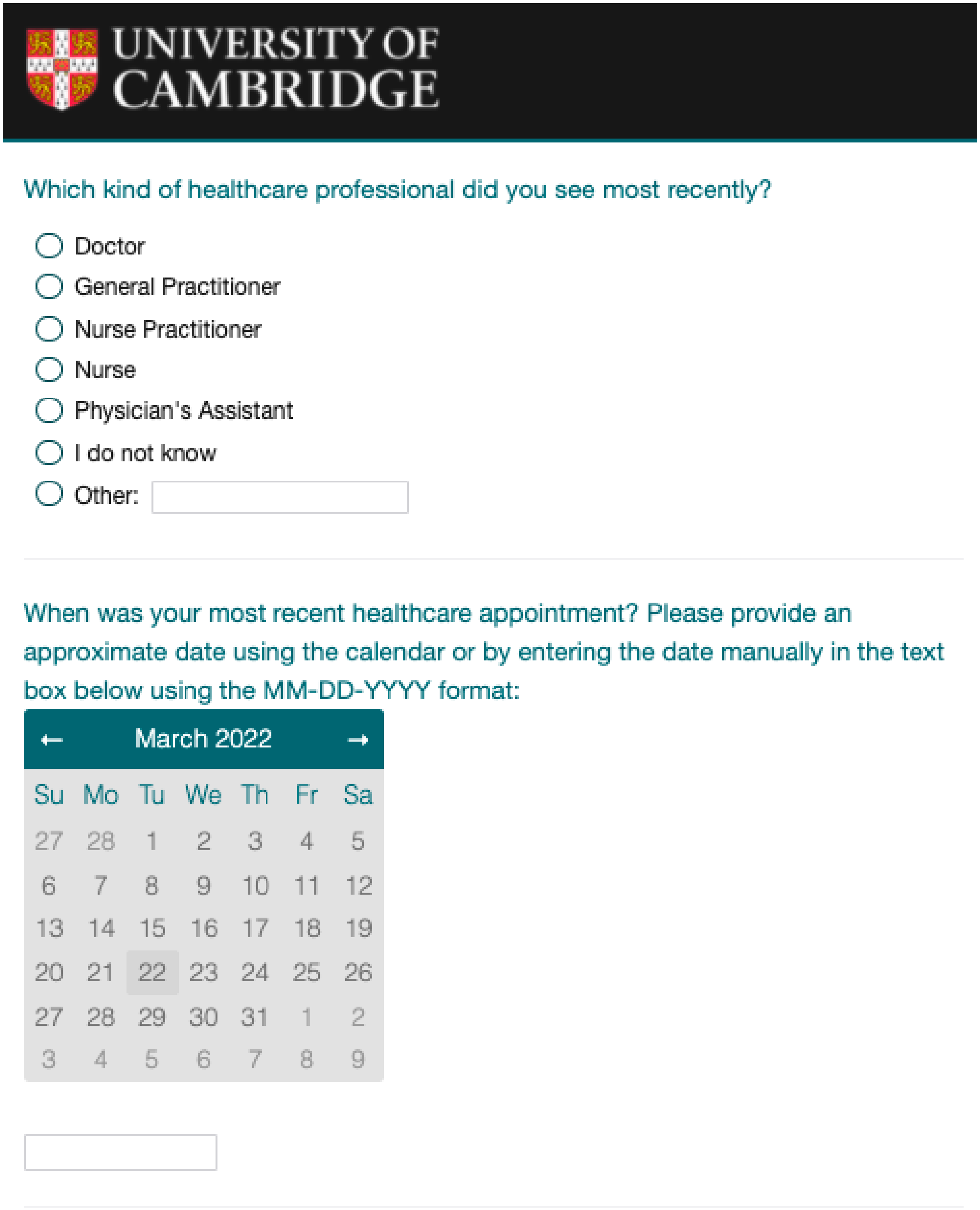

UNIVERSITY OF CAMBRIDGE

Which kind of healthcare professional did you see most recently?

- ◯ Doctor
- ◯ General Practitioner
- ◯ Nurse Practitioner
- ◯ Nurse
- ◯ Physician's Assistant
- ◯ I do not know
- ◯ Other: ________

When was your most recent healthcare appointment? Please provide an approximate date using the calendar or by entering the date manually in the text box below using the MM-DD-YYYY format:

← March 2022 →

Su	Mo	Tu	We	Th	Fr	Sa
27	28	1	2	3	4	5
6	7	8	9	10	11	12
13	14	15	16	17	18	19
20	21	22	23	24	25	26
27	28	29	30	31	1	2
3	4	5	6	7	8	9

Supplementary Figure S15: Content of Most Recent Healthcare Appointment

What was discussed in the healthcare appointment? Please select all that apply:

- ☐ One or more physical health conditions
- ☐ One or more mental health conditions
- ☐ Other (Please Specify): ______

Please answer the following questions about your experiences of going to see a healthcare professional (Doctor, General Practitioner, Nurse Practitioner, Nurse, or Physician's Assistant)

	Definitely Agree	Slightly Agree	Slightly Disagree	Definitely Disagree
I understood the questions my healthcare professional asked	○	○	○	○
The healthcare professional gave me enough time	○	○	○	○
The healthcare professional understood me when I described my symptoms	○	○	○	○
The healthcare professional attempted to help me with my symptoms	○	○	○	○
I do not think that the healthcare professional cared about my wellbeing	○	○	○	○

Supplementary Figure 16: Autism-Specific Healthcare From Main Healthcare Professional

UNIVERSITY OF CAMBRIDGE

Part Four: Autism and Healthcare

The following section will ask you questions about your healthcare experiences as an autistic individual.

Please answer the following questions about your experiences of going to see your main healthcare professional (Doctor, General Practitioner, Nurse Practitioner, Nurse, or Physician's Assistant)

	Definitely Agree	Slightly Agree	Slightly Disagree	Definitely Disagree
I have told my healthcare professional that I am autistic				
My healthcare professional and I have discussed my autism				
My healthcare professional knows what autism is				
I think that my healthcare professional usually tries to make adjustments for me because I am autistic				
I think that my healthcare professional usually considers my autism when making diagnoses and treatment plans				

Supplementary Table S1: Missing Data For Outcomes

	**Number of Autistic Participants with Missing Data N (%)**	**Number of Non-Autistic Participants with Missing Data N (%)**
Able to see healthcare professionals as often as they would like	5 (0.39)	1 (0.07)
Has health insurance or is part of a national healthcare program (e.g. NHS, Medicare, Medicaid, etc…)	27 (2.10)	43 (3.15)
Sensory Experience		
Reported at least one sensory difference (hyper- or hyposensitivity)	0	0
I am able to describe how my symptoms feel in my body	13 (1.01)	20 (1.47)
I am able to describe how bad my pain feels	12 (0.93)	20 (1.47)
I am able to describe my sensory processing differences to healthcare professionals *	103 (8.02)<br>17 (2.79) of eligible	772 (56.60)<br>17 (1.42) of eligible
The sensory environment of the waiting room is more overwhelming than other environments	13 (1.01)	25 (1.83)
The sensory environment of the office is more overwhelming than other environments	15 (1.17)	22 (1.61)
My senses frequently overwhelm me so that I have trouble focusing on conversations with healthcare professionals	13 (1.01)	21 (1.54)
Communication		
I am usually able to explain what my symptoms are	18 (1.40)	35 (2.57)
I usually understand what my healthcare professional means when they discuss my health	17 (1.32)	36 (2.71)
I do not usually ask all the questions I would like to about my health	20 (1.56)	41 (3.01)
I can bring up a health concern even if my healthcare professional doesn't ask about it	20 (1.56)	40 (2.93)
I know what is expected of me when I go to see my healthcare professional	17 (1.32)	37 (2.71)
Anxiety		
The idea of going to see a healthcare professional makes me feel anxious	24 (1.87)	47 (3.45)

The environment of the waiting room or office makes me feel anxious	26 (2.02)	48 (3.52)
I feel anxious when I see a different healthcare professional to whom I expect	27 (2.10)	50 (3.67)
The process of setting up an appointment makes me anxious	27 (2.10)	49 (3.59)
The process of picking up a prescription makes me anxious	30 (2.33)	51 (3.74)
I frequently leave my healthcare professional's office feeling as though I did not receive any help at all	26 (2.02)	49 (3.59)
Access and Advocacy		
Chosen not to go in to see a healthcare professional	29 (2.26)	70 (5.13)
I know who to contact if I have a healthcare concern	31 (2.41)	69 (5.06)
If I need to go see a healthcare professional, I am able to get there	34 (2.65)	69 (5.06)
I usually bring someone along to help support me in my appointments	31 (2.41)	71 (5.21)
If I need to go to the pharmacy, I am able to get there	32 (2.49)	70 (5.13)
I am able to follow a procedure for next steps if asked (for example, I will attend follow-up appointments, annual checkups if applicable, etc...)	33 (2.57)	71 (5.21)
I am able to make appointments for myself	32 (2.49)	74 (5.43)
I will wait until it is an emergency before I go to see a healthcare professional	31 (2.41)	72 (5.28)
System Problems		
In most appointments, I have enough time to discuss my concerns with healthcare professionals	43 (3.35)	103 (7.55)
If I need to go to see a specialist for a healthcare concern, I am able to do so	47 (3.66)	103 (7.55)
I often choose not to go to the doctor with concerns if I need to see a specialist because I know that it will take my many appointments before I can see the specialist	44 (3.42)	105 (7.70)
I usually leave my appointments knowing what the next steps are (i.e. follow-up appointments, medications, etc…)	45 (3.50)	108 (7.92)
I am provided with appropriate support after I receive a diagnosis of any kind (i.e. anything from infections to chronic conditions)	45 (3.50)	108 (7.92)
Triggers for a Shutdown or Meltdown		

The idea of going to see a healthcare professional	60 (4.67)	116 (8.50)
Setting up an appointment to see a healthcare professional	60 (4.67)	135 (9.90)
Sensory environment of the waiting room	61 (4.75)	128 (9.38)
Sensory environment of the office	66 (5.14)	132 (9.68)
Seeing a different healthcare professional to whom you expect	61 (4.75)	130 (9.53)
Talking to a healthcare professional	60 (4.67)	129 (9.46)
Picking up a prescription	62 (4.82)	135 (9.90)
Having to see many healthcare professionals before being able to talk to a specialist	57 (4.44)	129 (9.46)
After a diagnosis of any kind due to lack of follow-up or support	71 (5.53)	134 (9.82)

* only participants who had sensory differences were shown this question

Sensitivity Analysis Information 1: Healthcare Inequality Scores

We conducted a sensitivity analysis excluding all participants with missing data. For the majority of participants, they answered all 29 questions shown to them. However, the third question of the Sensory Sensitivity section (*"I am able to describe my sensory processing differences to healthcare professionals")* was only shown to the proportion of individuals who reported a sensory processing difficulty earlier in the survey. As such, there were only 28 questions shown to individuals without sensory processing differences. Thus, the sensitivity analysis below includes the 2,405 participants without missing data. The results did not change after running the results using this subsample; full results have been provided in Supplementary Tables S2 and S3.

Supplementary Table S2: Sensitivity Analysis of Healthcare Inequality Scores with Complete Cases Only

	**Unadjusted Model†**		**Adjusted Model††**	
	**OR (95% CI)**	**p-value**	**OR (95% CI)**	**p-value**
Sensory Sensitivity Section Score	14.462 (10.045, 21.032)	$< 2.22 \times 10^{-16}$	1.537 (1.440, 1.640)	$< 2.22 \times 10^{-16}$
Communication Section Score	19.253 (13.981, 26.737)	$< 2.22 \times 10^{-16}$	1.835 (1.710, 1.969)	$< 2.22 \times 10^{-16}$
Anxiety Section Score	21.929 (16.389, 29.567)	$< 2.22 \times 10^{-16}$	1.674 (1.590, 1.764)	$< 2.22 \times 10^{-16}$
Access and Advocacy Section Score	13.920 (8.499, 23.086)	$< 2.22 \times 10^{-16}$	1.426 (1.323, 1.538)	$< 2.22 \times 10^{-16}$
System Problems Score	10.768 (8.154, 14.298)	$< 2.22 \times 10^{-16}$	1.563 (1.474, 1.658)	$< 2.22 \times 10^{-16}$
Total Final Score	41.360 (24.185, 71.825)	$< 2.22 \times 10^{-16}$	1.133 (1.110, 1.156)	$< 2.22 \times 10^{-16}$
Assigned Female at Birth				
Sensory Sensitivity Section Score	22.405 (14.226, 35.890)	$< 2.22 \times 10^{-16}$	1.646 (1.518, 1.785)	$< 2.22 \times 10^{-16}$
Communication Section Score	31.079 (20.627, 47.531)	$< 2.22 \times 10^{-16}$	2.030 (1.855, 2.223)	$< 2.22 \times 10^{-16}$
Anxiety Section Score	46.027 (30.127, 71.606)	$< 2.22 \times 10^{-16}$	1.887 (1.750, 2.034)	$< 2.22 \times 10^{-16}$
Access and Advocacy Section Score	22.208 (11.782, 42.780)	$< 2.22 \times 10^{-16}$	1.531 (1.390, 1.687)	$< 2.22 \times 10^{-16}$
System Problems Score	16.160 (11.307, 23.328)	$< 2.22 \times 10^{-16}$	1.698 (1.574, 1.831)	$< 2.22 \times 10^{-16}$
Total Final Score	93.904 (47.026, 192.866)	$< 2.22 \times 10^{-16}$	1.165 (1.135, 1.195)	$< 2.22 \times 10^{-16}$
Assigned Male at Birth				
Sensory Sensitivity Section Score	8.816 (4.546, 17.654)	3.01×10^{-10}	1.406 (1.247, 1.585)	3.85×10^{-8}
Communication Section Score	8.879 (5.240, 15.338)	1.49×10^{-15}	1.543 (1.371, 1.736)	1.22×10^{-12}
Anxiety Section Score	13.038 (8.378, 20.594)	$< 2.22 \times 10^{-16}$	1.549 (1.427, 1.681)	$< 2.22 \times 10^{-16}$
Access and Advocacy Section Score	6.270 (2.867, 14.080)	5.91×10^{-6}	1.263 (1.114, 1.430)	2.62×10^{-4}
System Problems Score	5.871 (3.713 9.402)	7.71×10^{-14}	1.372 (1.242, 1.516)	6.79×10^{-10}
Total Final Score	17.238 (6.771, 45.847)	5.09×10^{-9}	1.095 (1.056, 1.135)	9.55×10^{-7}

† Binomial Logistic Regression (unadjusted)
†† Binomial Logistic Regression adjusting for age, ethnicity, education, and country of residence
OR = Odds Ratio
95% CI = 95% Confidence Interval
Sig. = Significance Level

Supplementary Table S3: Sensitivity Analysis of Healthcare Inequality Scores with Complete Cases Only

	Accuracy	Sensitivity	Specificity
Sensory Sensitivity Section Score	72.075	92.889	28.000
Communication Section Score	70.166	76.121	64.249
Anxiety Section Score	67.152	62.145	72.045
Access and Advocacy Section Score	57.640	66.632	48.760
System Problems Score	66.060	68.305	63.843
Total Final Score	70.340	83.814	41.608

Based on Unadjusted Binomial Logistic Regression analyses with autism diagnosis as the outcome variable

Sensitivity Analysis Information 2: Individuals Diagnosed with Anxiety

We also conducted a sensitivity analysis in which we controlled for previous diagnosis of Anxiety (using a binary measure of yes/no) for all questions relating to the Anxiety subsection, as we know that autistic individuals (and particularly autistic individuals AFAB) are more likely to have a diagnosis of anxiety compared to neurotypical peers.[4-6,8] While controlling for a diagnosis of anxiety did tamp down the magnitude of differences between autistic and non-autistic adults, autistic adults were still far more likely to report that specific circumstances induced anxiety across all measures (ORs: 2.2 – 4.6); full results of the sensitivity analysis can be found in Supplementary Table S4.

Supplementary Table S4: Sensitivity Analysis for Individuals with Anxiety

	Adjusted Model†		
	OR (95% CI)	p-value	Sig.
The idea of going to see a healthcare professional makes me feel anxious	2.236 (1.827, 2.735)	7.55×10^{-15}	***
The environment of the waiting room or office makes me feel anxious	3.508 (2.895, 4.252)	$< 2.22 \times 10^{-16}$	***
I feel anxious when I see a different healthcare professional to whom I expect	4.575 (3.737, 5.601)	$< 2.22 \times 10^{-16}$	***
The process of setting up an appointment makes me anxious	3.576 (2.916, 4.384)	$< 2.22 \times 10^{-16}$	***
The process of picking up a prescription makes me anxious	3.280 (2.718, 3.958)	$< 2.22 \times 10^{-16}$	***
I frequently leave my healthcare professional's office feeling as though I did not receive any help at all	2.725 (2.283, 3.252)	$< 2.22 \times 10^{-16}$	***

† Binomial Logistic Regression adjusting for age, ethnicity, education, country of residence, and diagnosis of Anxiety Disorder
OR = Odds Ratio
95% CI = 95% Confidence Interval
Sig. = Significance Level

Supplementary Table S5: Participant Demographics for Covid Analyses

Characteristics	Autism (n = 1,149)	Controls (n = 1,136)	p-values (Sig.)
Age (years), mean (SD)	41.49 (14.45)	39.39 (16.11)	5.68×10^{-5} (***)
Age (years), categories, N (%)			
16-29	296 (25.76)	388 (34.16)	
30-39	240 (20.89)	221 (19.45)	
40-49	246 (21.41)	203 (17.87)	
50-59	230 (20.02)	172 (15.14)	
60-69	104 (9.05)	102 (8.98)	
70+	33 (2.87)	50 (4.40)	
AQ-10 Score, mean (SD)	8.07 (1.88)	3.76 (2.64)	$< 2.22 \times 10^{-16}$ (***)
Female	8.17 (1.73)	3.65 (2.66)	$< 2.22 \times 10^{-16}$ (***)
Male	7.89 (2.11)	3.96 (2.58)	$< 2.22 \times 10^{-16}$ (***)
Other	8.75 (1.89)	--	
Sex Assigned at Birth, N (%)			0.135
Female	737 (64.14)	736 (64.79)	
Male	408 (35.51)	400 (35.21)	
Other	4 (0.35)	0	
Ethnicity, N (%)			4.62×10^{-7} (***)
White	994 (86.51)	897 (78.96)	
Non-White	149 (12.97)	238 (20.95)	
African	3 (0.26)	10 (0.88)	
Arab	0	5 (0.44)	
Caribbean	6 (0.52)	1 (0.09)	
Hispanic	9 (0.78)	30 (2.64)	
Jewish	22 (1.92)	33 (2.90)	
Mixed Race	73 (6.35)	102 (8.98)	
Turkish	1 (0.09)	7 (0.62)	
Other	35 (3.05)	50 (4.40)	
Missing	6 (0.52)	1 (0.09)	
Education, N (%)			2.97×10^{-5} (***)
No formal qualifications	50 (4.35)	24 (2.11)	
Further vocational qualifications	181 (15.75)	132 (11.61)	
Secondary School/ High School	197 (17.15)	190 (16.73)	
University Undergraduate	358 (31.16)	342 (30.11)	
University Postgraduate	363 (31.59)	448 (39.44)	
Country of Residence			$< 2.22 \times 10^{-16}$ (***)
Australia	16 (1.39)	32 (2.82)	
Canada	38 (3.31)	42 (3.70)	
Germany	34 (2.96)	25 (2.20)	
Netherlands	23 (2.00)	29 (2.55)	
United Kingdom	751 (65.36)	502 (44.19)	
United States	135 (11.75)	138 (12.15)	
Other	151 (13.14)	366 (32.22)	
Missing	1 (0.09)	2 (0.17)	

p-values were from Pearson's Chi Square test (categorical) or from a Mann-Whitney U test (means)
SD = standard deviation
Sig. = significance level

Supplementary Table S6: No Significant Differences in Healthcare Quality for Autistic or Non-autistic Adults Before and After the Onset of the Pandemic

	Pre-Pandemic N (%)	Post-Pandemic N (%)	Odds Ratio (95% CI)	p-value	Sig.
Autistic Participants					
I understood the questions my healthcare professional asked	777 (88.60)	242 (88.97)	1.038 (0.665, 1.660)	0.913	
The healthcare professional gave me enough time	630 (71.84)	200 (73.53)	1.089 (0.794, 1.503)	0.642	
The healthcare professional understood me when I described my symptoms	616 (70.24)	196 (72.06)	1.093 (0.801, 1.500)	0.594	
The healthcare professional attempted to help me with my symptoms	675 (76.97)	211 (77.57)	1.035 (0.741, 1.459)	0.869	
I do not think that the healthcare professional cared about my wellbeing	224 (25.54)	54 (19.85)	0.722 (0.507, 1.018)	0.062	
Non-Autistic Participants					
I understood the questions my healthcare professional asked	700 (96.82)	401 (97.09)	1.098 (0.518, 2.449)	0.860	
The healthcare professional gave me enough time	617 (85.34)	363 (87.89)	1.247 (0.860, 1.828)	0.245	
The healthcare professional understood me when I described my symptoms	628 (86.86)	369 (89.35)	1.268 (0.857, 1.901)	0.259	
The healthcare professional attempted to help me with my symptoms	629 (87.00)	377 (91.28)	1.564 (1.031, 2.417)	0.033	
I do not think that the healthcare professional cared about my wellbeing	135 (18.67)	79 (19.13)	1.030 (0.746, 1.416)	0.875	

95% CI = 95% Confidence Interval
Sig. = Significance Level

Supplementary Table S7: Full Results for Binomial Logistic Regression Predicting Autism Status from Health Inequality Scores

	Detailed Adjusted Models	
	**OR (95% CI)**	**p-value**
Sensory Sensitivity Section Score	**1.548 (1.454, 1.649)**	**$< 2.22 \times 10^{-16}$**
Age	**1.012 (1.003, 1.020)**	**5.87×10^{-3}**
Ethnicity	0.735 (0.545, 0.991)	0.043
Education (Further vocational qualifications)	0.576 (0.273, 1.219)	0.149
Education (Secondary School)	0.711 (0.343, 1.475)	0.360
Education (University qualifications)	0.558 (0.272, 1.143)	0.111
Education (Postgraduate qualifications)	0.680 (0.332, 1.392)	0.291
Country (Australia)	0.329 (0.151, 0.719)	5.32×10^{-3}
Country (Canada)	0.767 (0.428, 1.373)	0.371
Country (Germany)	0.909 (0.480, 1.722)	0.770
Country (Netherlands)	0.748 (0.364, 1.539)	0.430
Country (Other)	**0.337 (0.252, 0.452)**	**4.95×10^{-13}**
Country (United States of America)	0.587 (0.417, 0.826)	2.25×10^{-3}
Communication Section Score	**1.822 (1.702, 1.950)**	**$< 2.22 \times 10^{-16}$**
Age	**1.016 (1.009, 1.022)**	**2.41×10^{-6}**
Ethnicity	0.868 (0.679, 1.110)	0.258
Education (Further vocational qualifications)	0.567 (0.313, 1.028)	0.062
Education (Secondary School)	0.618 (0.347, 1.099)	0.101
Education (University qualifications)	0.476 (0.269, 0.839)	0.010
Education (Postgraduate qualifications)	0.598 (0.339, 1.054)	0.075
Country (Australia)	0.392 (0.210, 0.733)	3.38×10^{-3}
Country (Canada)	0.653 (0.412, 1.035)	0.069
Country (Germany)	1.025 (0.593, 1.771)	0.929
Country (Netherlands)	0.768 (0.427, 1.382)	0.378
Country (Other)	**0.323 (0.254, 0.410)**	**$< 2.22 \times 10^{-16}$**
Country (United States of America)	0.749 (0.567, 0.990)	0.042
Anxiety Section Score	**1.679 (1.596, 1.767)**	**$< 2.22 \times 10^{-16}$**
Age	**1.017 (1.010, 1.023)**	**1.29×10^{-6}**
Ethnicity	0.878 (1.010, 1.023)	0.318
Education (Further vocational qualifications)	0.495 (0.680, 1.133)	0.020
Education (Secondary School)	0.549 (0.273, 0.897)	0.041
Education (University qualifications)	0.405 (0.309, 0.975)	1.72×10^{-3}
Education (Postgraduate qualifications)	0.528 (0.230, 0.713)	0.026
Country (Australia)	0.353 (0.300, 0.927)	1.32×10^{-3}
Country (Canada)	0.666 (0.187, 0.666)	0.089
Country (Germany)	1.122 (0.416, 1.064)	0.685
Country (Netherlands)	0.778 (0.642, 1.964)	0.424
Country (Other)	**0.355 (0.278, 0.454)**	**2.22×10^{-16}**
Country (United States of America)	0.666 (0.500, 0.887)	5.42×10^{-3}
Access and Advocacy Section Score	**1.412 (1.313, 1.519)**	**$< 2.22 \times 10^{-16}$**
Age	1.006 (1.000, 1.013)	0.039
Ethnicity	0.772 (0.608, 0.979)	0.033
Education (Further vocational qualifications)	0.469 (0.267, 0.821)	8.02×10^{-3}
Education (Secondary School)	0.479 (0.279, 0.824)	7.83×10^{-3}
Education (University qualifications)	**0.340 (0.199, 0.579)**	**7.43×10^{-5}**
Education (Postgraduate qualifications)	0.436 (0.256, 0.742)	2.21×10^{-3}

Country (Australia)	0.377 (0.208, 0.683)	1.31×10^{-3}
Country (Canada)	0.567 (0.364, 0.882)	0.012
Country (Germany)	0.966 (0.572, 1.630)	0.897
Country (Netherlands)	0.595 (0.340, 1.042)	0.069
Country (Other)	**0.286 (0.227, 0.360)**	**$< 2.22 \times 10^{-16}$**
Country (United States of America)	0.739 (0.567, 0.964)	0.026
System Problems Score	**1.568 (1.479, 1.662)**	**$< 2.22 \times 10^{-16}$**
Age	1.010 (1.004, 1.017)	1.77×10^{-3}
Ethnicity	0.849 (0.661, 1.092)	0.202
Education (Further vocational qualifications)	**0.362 (0.201, 0.651)**	**6.94×10^{-4}**
Education (Secondary School)	0.430 (0.244, 0.758)	3.55×10^{-3}
Education (University qualifications)	**0.281 (0.161, 0.491)**	**8.21×10^{-6}**
Education (Postgraduate qualifications)	**0.368 (0.211, 0.642)**	**4.37×10^{-4}**
Country (Australia)	0.376 (0.201, 0.702)	2.17×10^{-3}
Country (Canada)	0.665 (0.419, 1.053)	0.082
Country (Germany)	1.171 (0.673, 2.040)	0.576
Country (Netherlands)	0.724 (0.403, 1.300)	0.279
Country (Other)	**0.360 (**0.283, 0.459)	**2.22×10^{-16}**
Country (United States of America)	0.890 (0.674, 1.174)	0.408
Total Final Score	**1.133 (1.110, 1.156)**	**$< 2.22 \times 10^{-16}$**
Age	1.013 (1.005, 1.156)	2.87×10^{-3}
Ethnicity	0.815 (0.595, 1.115)	0.200
Education (Further vocational qualifications)	0.523 (0.241, 1.134)	0.101
Education (Secondary School)	0.696 (0.326, 1.485)	0.349
Education (University qualifications)	0.565 (0.268, 1.191)	0.134
Education (Postgraduate qualifications)	0.650 (0.310, 1.366)	0.255
Country (Australia)	0.348 (0.157, 0.769)	9.15×10^{-3}
Country (Canada)	0.693 (0.390, 1.233)	0.212
Country (Germany)	0.919 (0.471, 1.794)	0.804
Country (Netherlands)	0.853 (0.406, 1.793)	0.675
Country (Other)	**0.368 (0.271, 0.498)**	**1.24×10^{-10}**
Country (United States of America)	0.669 (0.473, 0.946)	0.023
